# HSP90 regulates dCK stability and inhibits ionizing radiation-induced ferroptosis in cervical cancer cells

**DOI:** 10.1038/s41420-025-02388-x

**Published:** 2025-04-22

**Authors:** Yue Wang, Huilin Ji, Tianpeng Yang, Yi Liu, Xiang He, Xinyue Jiang, Zipeng Lu, Liu Han, Xiaodong Liu, Shumei Ma

**Affiliations:** 1 School of Public Health, Wenzhou Medical University, Wenzhou, China; 2 South Zhejiang Institute of Radiation Medicine and Nuclear Technology, Wenzhou, China

**Keywords:** Cell death, Radiotherapy

## Abstract

Cervical squamous cell carcinoma (CESC) is one of the most common cancers in women, and radiotherapy has been used as a primary treatment. However, its efficacy is limited by intrinsic and acquired radiation resistance. Our previous study demonstrated that Deoxycytidine kinase (dCK) inhibits ionizing radiation (IR)-induced cell death, including apoptosis and mitotic catastrophe, and dCK is a HSP90-interacting protein by mass spectrometry and co-immunoprecipitation assay. In the present study, we found that dCK inhibited IR-induced ferroptosis by increasing the activity and stability of SLC7A11. Using the E3 ubiquitin ligase database (UbiBrowser), we predicted NEDD4L as a potential ubiquitin ligase of dCK, and WWP1/2 as potential ubiquitin ligases of NEDD4L, respectively. These predictions were subsequently verified through a ubiquitination IP assay. Our findings indicate that HSP90 regulates dCK stability by inhibiting NEDD4L through the recruitment of ubiquitin ligases WWP1/2. In summary, our study reveals the HSP90-WWP1/WWP2-NEDD4L-dCK-SLC7A11 axis as a critical regulator of IR-induced ferroptosis in HeLa cells. These findings provide valuable insights into potential strategies for the radiosensitization of cervical cancer.

## Introduction

Cervical squamous cell carcinoma (CESC) is the second leading cause of death from cancer among childbearing women worldwide [1].Radiotherapy has been used as a primary treatment for cervical cancer, especially for locally advanced cases [2–4]. However, its intrinsic and acquired radiation resistance lead to a poor prognosis, posing an urgent problem in clinical settings. As a cytotoxic therapy, ionizing radiation (IR) exerts its therapeutic effect by inducing DNA double-strand breaks (DSBs) in tumor cells [5]. When exposed to high-energy IR, the tumor cells are destroyed, leading to various types of cell death [6, 7]. Ferroptosis is a recently identified iron-dependent form of non-apoptotic cell death, characterized by the buildup of cytotoxic lipid peroxides [8]. Many studies have shown that ferroptosis holds promise for cancer treatments such as radiotherapy. However, the relationship between radiotherapy and ferroptosis in cervical cancer remains undefined.

Deoxycytidine kinase (dCK) is one of the four principal salvage enzymes in mammalian cells. It is responsible for phosphorylating 2′-deoxycytidine, 2′-deoxyadenosine and 2′-deoxyguanosine to their corresponding monophosphorylated forms [9], providing deoxynucleotide triphosphates for DNA replication and repair [10]. In addition, it also acts as a critical enzyme required for the anti-tumor activity of many nucleoside analogs [11]. Nucleoside analogs have been frequently used in combination with radiotherapy, and most of those currently employed are primarily activated by dCK [12].

In this study, we found that dCK knockdown could enhance ferroptosis and radiosensitivity of HeLa cells. We demonstrated for the first time that HSP90 was a novel binding partner of dCK, which promoted the dCK protein stability by regulating WWP1/WWP2-NEDD4L. These findings provide valuable insights into potential strategies for the radiosensitization of cervical cancer.

## Results

### dCK knockdown promoted radiosensitivity of HeLa cells

**Fig. 1:**
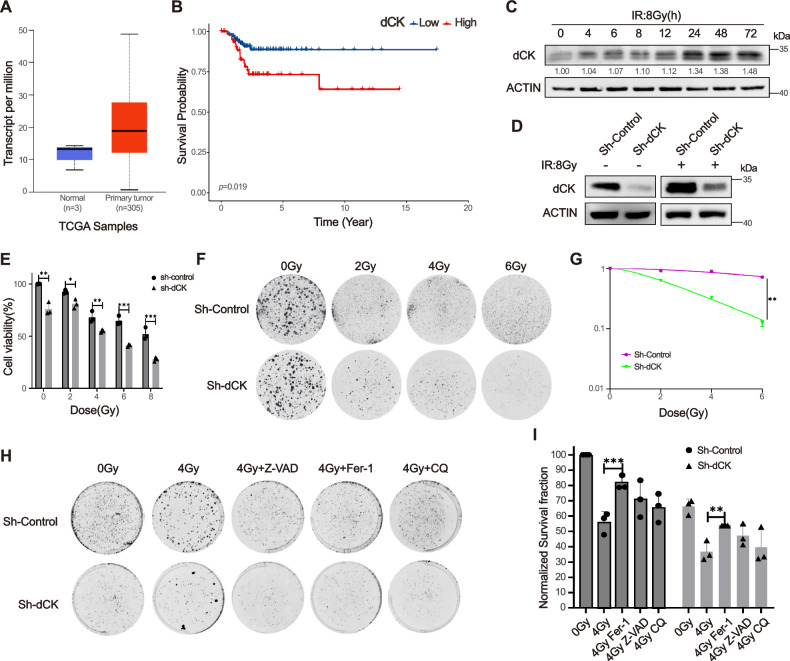
dCK knockdown promoted radiosensitivity of HeLa cells. **A** Comparison of dCK expression levels between CESC tumors and normal tissues. **B** Kaplan–Meier curves of RFS in cervical cancer according to the dCK expression levels. **C** The expression level of dCK in HeLa cells after 8 Gy of IR in different time. **D** The knockdown efficiency of dCK was determined by Western blot in HeLa cells at 48 h after exposure to 8 Gy of IR. **E** The cell viability of sh-Control and sh-dCK HeLa cells after IR were detected by CCK-8 assay. **F** The radiosensitivity of sh-Control and sh-dCK HeLa cells were detected by colony formation assay. **G** Survival fraction with multi-target single-hit model. **(H-I)** Colony formation assay in sh-Control and sh-dCK HeLa cells that were pretreated with 20 μM ferrostatin-1 (fer-1), 20 μM Z-VAD-fmk (Z-VAD) or 10 μM chloroquine (CQ) or DMSO for 2 h after 4 Gy of IR. Error bars are means ± SD, *n* = 3 independent repeats. ns, non significance; *p < 0.05; **p < 0.01; ***p < 0.001. *P* values were calculated by using two-tailed unpaired student’s *t*-test.

We analyzed the expression level of dCK in The Cancer Genome Atlas (TCGA) database, and verified that dCK was significantly increased in CESC tissues (Fig. 1A). And high levels of dCK were correlated with poor prognosis of CESC patients as revealed by Kaplan-Meier curves (https://kmplot.com) (Fig. 1B). The results showed a time-dependent upregulation of dCK (Fig. 1C). Consequently, we constructed a stable knockdown HeLa cell model by transfected with dCK shRNA, and examined the transfection efficiency (Fig. 1D). After IR exposure, the cell death rate of dCK knockdown cells were significantly increased at 48 h (Fig. S1). Furthermore, dCK knockdown significantly inhibited the proliferation of HeLa cells exposed to IR (Fig. 1E). Consistently, colony formation assay showed that dCK knockdown increased the radiosensitivity in HeLa cells (Fig. 1F, G). Taken together, these results demonstrated dCK knockdown promoted radiation-induced cell death and enhanced radiosensitivity of HeLa cells.

### dCK knockdown promoted radiation-induced ferroptosis in HeLa cells

It is commonly known that IR can cause various forms of cell death. Different cell death inhibitors, such as those that inhibit apoptosis (Z-VAD), ferroptosis (Fer-1) and autophagy (CQ), were applied to dCK knockdown HeLa cells in order to ascertain the type of cell death that was induced after IR treatment. The cells were then treated with 4 Gy of IR. Fer-1 attenuated IR-induced cell death, indicating that dCK knockdown may facilitate radiation-induced ferroptosis (Fig. 1H, I).

**Fig. 2:**
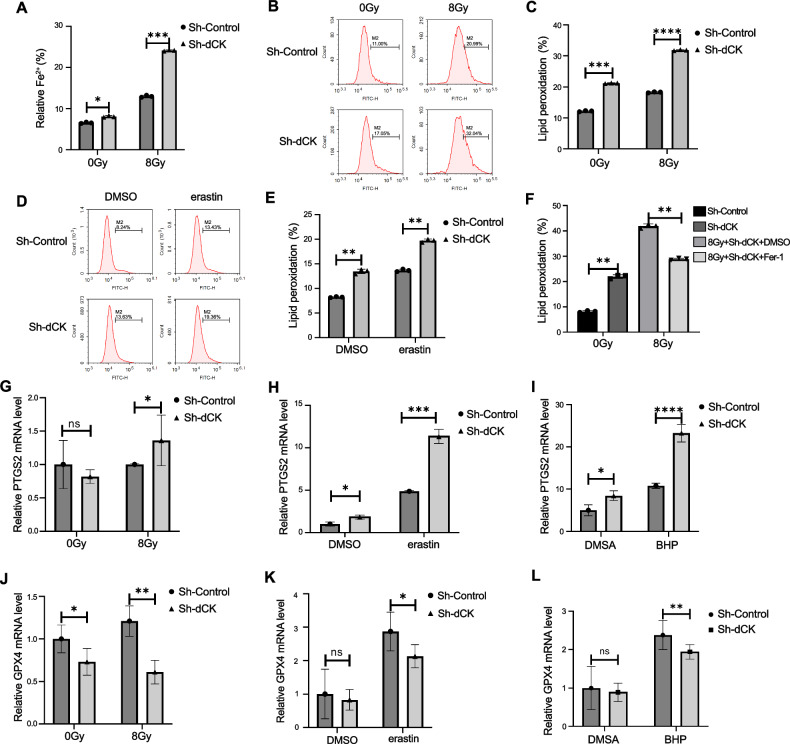
dCK knockdown promoted radiation-induced ferroptosis. **A** Fe^2+^ concentrations in sh-dCK and sh-Control HeLa cells were found using the Phen Green^TM^SK staining method. **B**–**E** Lipid peroxidation measurement in sh-Control and sh-dCK HeLa cells 48 h following 8 Gy of infrared radiation (B-C) and erastin or DMSO treatment (**D**, **E**). **F** Evaluation of lipid peroxidation in sh-Control and sh-dCK HeLa cells treated with 20 μM ferrostatin-1 or DMSO for 48 h prior to being exposed to an 8 Gy dose of IR. PTGS2 expression in sh-Control and sh-dCK HeLa cells was analyzed using qRT-PCR 48 h after the cells were exposed to 8 Gy of IR (**G**), erastin (**H**), and t-BHP (**I**). GPX4 expression in sh-Control and sh-dCK was examined using qRT-PCR. HeLa cells 48 h after being exposed to 8 Gy of IR (**J**), erastin (**K**), and t-BHP (**L**). The control groups received DMSO treatment for the same amount of time. The error bars represent means ± SD based on three independent repeats. * Denotes non-significance; **p < 0.01; ***p < 0.001. Using a two-tailed unpaired student’s *t*-test, *P* values were determined.

We ran a number of tests to confirm whether or not dCK knockdown promotes radiation-induced ferroptosis. Fenton reactions depend on ferroptosis, which is indicated by the presence of Fe^2+^. As anticipated, dCK knockdown HeLa cells had a significantly higher intracellular Fe^2+^ concentration than the control group; this effect may be amplified by IR treatment (Fig. 2A). Then, compared with the shControl, lipid peroxidation detected by BODIPY C11 staining was significantly increased in dCK knockdown HeLa cells after IR or erastin treatment (Fig. 2B–E), which could be reversed by Fer-1 (Fig. 2F). Additionally, we found the mRNA expression of PTGS2, a marker of ferroptosis, was increased in dCK knockdown HeLa cells after IR treatment (Fig. 2G), and the elevation was also observed in ferroptosis inducer Erastin (Fig. 2H) and oxidative stress inducer tert-butyl hydroperoxide (t-BHP) (Fig. 2I) treated cells. Similarly, the mRNA levels of GPX4 were decreased (Fig. 2J–L). These findings suggested that radiation-induced ferroptosis was aided by dCK knockdown.

### dCK increased the mRNA and protein level of SLC7A11

**Fig. 3:**
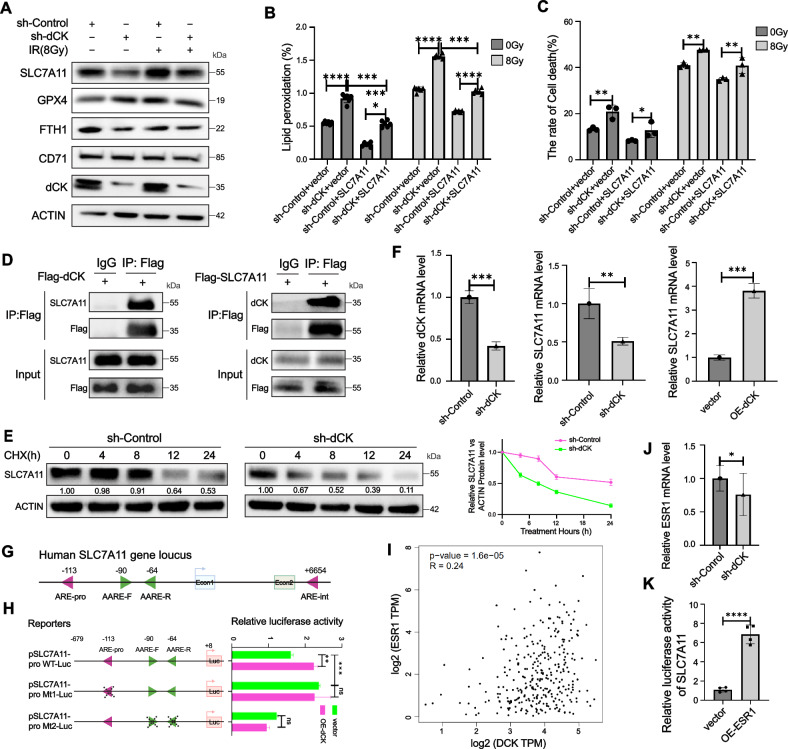
dCK regulated the mRNA level and protein stability of SLC7A11. **A** Western blotting in HeLa cells with sh-Control and sh-dCK. **B** Evaluation of SLC7A11-expressing sh-Control and sh-dCK HeLa cells’ lipid peroxidation. **C** Evaluation of cell death in HeLa cells expressing sh-Control and sh-dCK and expressing SLC7A11. **D** Western blot analysis to identify the dCK and SLC7A11 interaction. **E** After cycloheximide (CHX) treatment, the half-life of the endogenous SLC7A11 protein, which had been lowered by dCK knockdown, was examined. **F** HeLa cells with dCK knockdown and overexpression subjected to qRT-PCR analysis. **G**, **H** An illustration of the human SLC7A11 gene schema. The purple and green triangles, respectively, represent the two AAREs (AARE-F and AARE-R) and the putative AREs (ARE-pro and ARE-int). **I** Scatter plots for TCGA data correlation analysis between ESR1 and dCK. **J** qRT-PCR analysis in HeLa cells with sh-Control and sh-dCK. **K** The effect of ESR1 overexpression on the promoter activity of SLC7A11 was investigated using the promoter dual-luciferase reporter assay.The error bars represent means ± SD based on three independent repeats. * Denotes non-significance; **p < 0.01; ***p < 0.001. Using a two-tailed unpaired student’s *t*-test, *P* values were determined.

In order to investigate the fundamental mechanism by which dCK regulates ferroptosis in HeLa cells, we found that the expression of multiple ferroptosis-related proteins, most notably SLC7A11, was downregulated in dCK knockdown cells (Fig. 3A). The degree of lipid peroxidation and the rate of cell death verified SLC7A11’s critical function in dCK-mediated ferroptosis (Figs. 3B, C and S2). The interactions between dCK and SLC7A11 were verified by IP in order to comprehend how dCK may regulate the expression of SLC7A11 (Fig. 3D). We looked at how dCK affected SLC7A11 stability in HeLa cells. To prevent SLC7A11 protein production, cycloheximide (CHX), an inhibitor of protein synthesis, was used. dCK knockdown increased the rate of protein breakdown by reducing the half-life of the SLC7A11 protein (Fig. 3E). Remarkably, RT-qPCR data revealed stable patterns in SLC7A11 and dCK mRNA levels, regardless of dCK overexpression or knockdown (Fig. 3F). Therefore, it is possible that dCK prevents radiation-induced ferroptosis in HeLa cells via boosting SLC7A11 transcriptional activity. Two amino acid response elements (AAREs) and an ARE have been found in mouse SLC7A11, which are in charge of SLC7A11 gene expression [13]. To be able to achieve this, we created a set of human-derived SLC7A11 gene promoter luciferase vectors that include the wild-type, AAR, and AARE mutants. The luciferase reporter test confirmed that dCK could raise SLC7A11wild type, but not mutant, promoter activity (Fig. 3G, H). Next, using the online transcription factor prediction tool hTFtarget, we identified ESR1, which was identified as a potential target gene for SLC7A11. In the meantime, our earlier research has demonstrated that ESR1 was a transcription factor for SLC7A11 and that it could control SLC7A11 transcription [14]. Therefore, we looked at whether dCK affected the ESR1/SLC7A11 pathway, which in turn affects SLC7A11 transcriptional activity in HeLa cells. A positive connection between dCK and ESR1 in cervical cancer was found using correlation analysis (Fig. 3I). Furthermore, ESR1 mRNA expression was shown to be reduced by dCK knockdown, according to RT-qPCR data (Fig. 3J). SLC7A11 was confirmed to be a direct target gene of ESR1 by the dual-luciferase reporter experiment (Fig. 3K). These findings showed that transcription factor ESR1 was involved in the regulation of SLC7A11 transcription by dCK. All things considered, our findings showed that SLC7A11 expression was upregulated at both the transcriptional and post-transcriptional levels as a result of dCK knockdown, which exacerbated radiation-induced ferroptosis.

### The protein stability of dCK was regulated by HSP90 via ubiquitin-proteasome pathway in HeLa cells

**Fig. 4:**
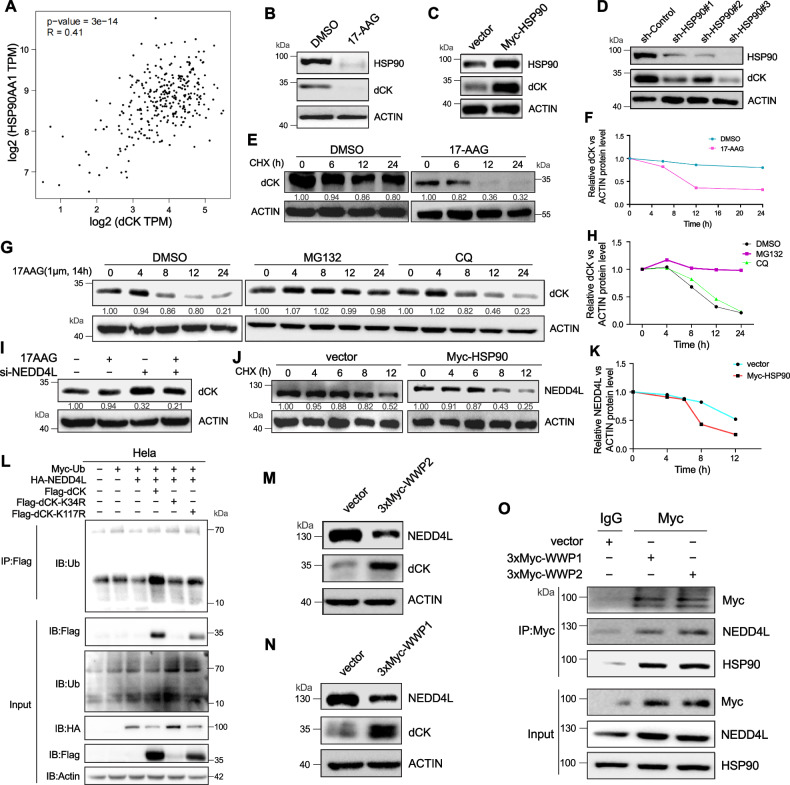
The protein stability of dCK was regulated by HSP90 via ubiquitin-proteasome pathway in HeLa cells. **A** Scatter plots for TCGA data’s HSP90AA1 and dCK correlation analysis. **B** 17-AAG lowered the levels of dCK and endogenous HSP90 protein. **C** Western blot analysis in HeLa cells overexpressing HSP90. **D** HeLa cells transfected with sh-Control, sh-HSP90#1, sh-HSP90#2, and sh-HSP90#3 were subjected to Western blot analysis. **E**, **F** Analysis was done on the endogenous dCK protein half-life that decreased by 17-AAG after cycloheximide (CHX) treatment. **G**, **H** HeLa cells were treated with 10 µM MG-132 and 30 μM CQ to prevent proteasomal degradation and autophagosome-lysosome fusion. **I** Western blotting was used to evaluate the expression of dCK in HeLa cells following either si-NEDD4L alone or in combination with 17-AAG. **J**, **K** The analysis of the experiment’s half-life of the endogenous NEDD4L protein was conducted after 48 h of transfection with Myc-HSP90 plasmids, and the sample was treated with cycloheximide (CHX) for 4 6, 8, 12 h. **L** In HeLa cells, the input and ubiquitin IP samples were subjected to Western blot analysis using the designated antibody. **M**, **N** Western blot analysis of NEDD4L and dCK protein level. **O** The interaction between WWP1/2 and NEDD4L, dCK or HSP90.

Our earlier study established that dCK was one of the HSP90 substrate proteins. Correlation analysis also revealed a positive correlation between dCK and HSP90AA1 (Fig. 4A). Tanespimycin (17-AAG), an HSP90 inhibitor, also entered our research and has been shown to work in concert with a number of clinical anticancer medications, including UCN-01, etoposide, and cisplatins [15]. When 17-AAG was given to HeLa cells to examine whether HSP90 regulated dCK, we discovered that both dCK and HSP90 expression were downregulated (Fig. 4B). Similarly, overexpression of HSP90 resulted in enhanced dCK expression(Fig. 4C). Next, we used HSP90 shRNA transfection to create stable knockdown HeLa cell models (Fig. 4D), In order to ascertain the precise mechanism by which HSP90 knockdown negatively regulates dCK expression in HeLa cells, dCK protein stability was assessed in HeLa cells treated with 17-AAG and CHX. HSP90 knockdown resulted in a shorter half-life of the dCK protein, as seen in (Fig. 4E, F). Additionally, CQ and MG132 were employed to test whether the protein degradation is mediated by ubiquitin-proteasome or autophagy-lysosome pathway. The results showed that HSP90 knockdown influenced dCK protein stability by boosting dCK ubiquitination and degradation (Fig. 4G, H). Above all, we discovered that HSP90 may control the radiosensitivity of HeLa cells by influencing the breakdown of the dCK protein.

### NEDD4L was inhibited by HSP90 through recruitment of the ubiquitin ligase WWP1/2, which then regulated the stability of dCK

We examined via UbiBrowser for binding partners to determine which E3 ubiquitin-ligases cause dCK ubiquitination (Fig. S3A–C). NEDD4L was chosen for further investigation. After transfecting HeLa cells with NEDD4L-siRNA, we observed a rise in the quantity of dCK protein by western blot. Also, it is possible for the inhibition of dCK by 17-AAG to partially reappear (Fig. 4I). HSP90 overexpression decreased the half-life of NEDD4L, as (Fig. 4J, K) illustrates. Protein co-immunoprecipitation (Co-IP) showed a link between dCK and NEDD4L. More precisely, as shown in Fig. 4L, greater contact improved dCK ubiquitination. To further explore the mechanism involved, we predicted the potential binding sites of NEDD4L and dCK by UniBrowser and constructed the corresponding K-R mutants, and the experimental results showed that NEDD4L mainly binds to the K34 site of dCK, thus mediating the ubiquitination of dCK. It was unclear, meanwhile, how HSP90 and NEDD4L were regulated. The E3 ubiquitin-ligase of NEDD4L was screened out using the same techniques and protocol (Fig. S3D–F). After careful study, we ultimately decided to use WWP1/2, which are members of the NEDD4 family, as research objectives. The overexpression of WWP1/2 was shown to be required for the degradation of NEDD4L protein, as evidenced by the considerable rise in dCK protein level and decrease in NEDD4L protein level (Fig. 4M, N). The Co-IP that followed demonstrated how NEDD4L and WWP1/2 interacted. Remarkably, Co-IP also revealed a robust interaction between WWP1/2 and HSP90 (Fig. 4O). Consistently, fluorescence co-localisation experiments confirmed that HSP90, WWP1, NEDD4L and dCK bind to each other in the cytoplasm (Fig. S4). When combined, these findings suggested that the dCK protein stability was regulated via the HSP90-WWP1/2-NEDD4L axis.

### HSP90-WWP1/2 -NEDD4L-dCK regulated radiation-induced ferroptosis of HeLa cells

**Fig. 5:**
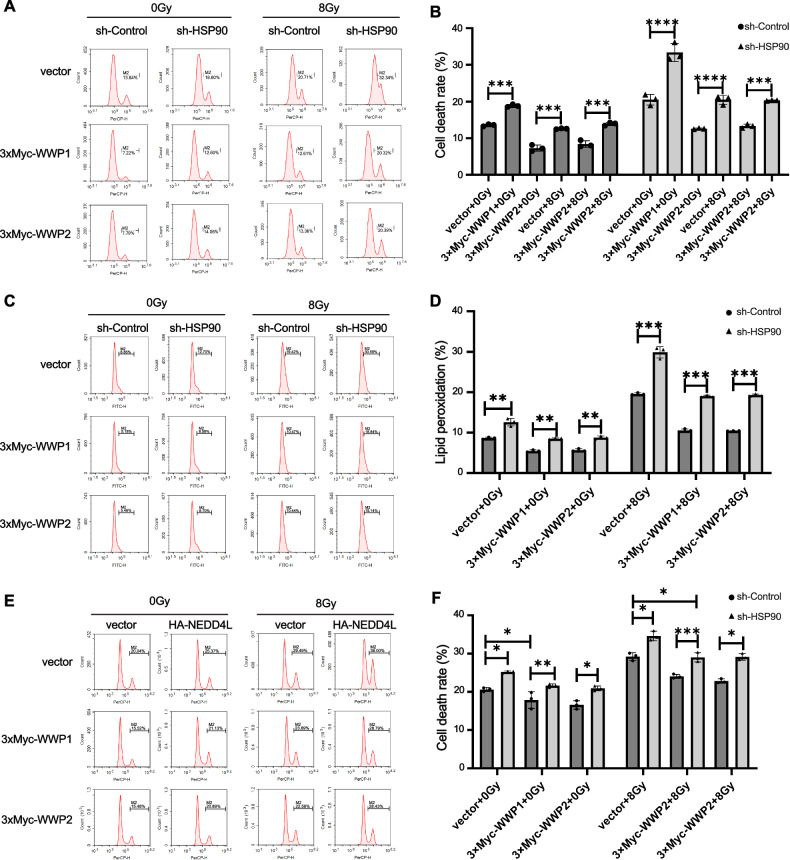
HSP90-WWP1/WWP2-NEDD4L axis regulated radiation-induced ferroptosis of HeLa cells. **A**, **B** Evaluation of cell death in sh-Control and sh-HSP90 HeLa cells expressing WWP1/2 48 h after 8 Gy of IR irradiation. **C**, **D** Evaluation of lipid peroxidation in sh-Control and sh-HSP90 HeLa cells expressing WWP1/2 48 h after 8 Gy of IR exposure. **E**, **F** Analysis of cell death rate in co-expressing WWP1/2 and NEDD4L in HeLa cells 48 h following 8 Gy of IR irradiation. The error bars show the means ± SD from three separate repeats. *p* < 0.05 for *, *p* < 0.01 for **, and *p* < 0.001 for ***. Using a two-tailed unpaired student’s *t*-test, *P* values were determined.

We then wanted to explore whether HSP90-WWP1/2-NEDD4L axis could play a role in radiation-induced ferroptosis. It is showed that exogenous WWP1/2 significantly increased the cell death as well as lipid peroxidation in shHSP90 cells, and IR-treatment could amplify therapeutic effects (Fig. 5A–D). We also found that exogenous WWP1/2 expression decreased cell death in HeLa cells, while exogenous NEDD4L expression could reverse this (Fig. 5E, F). These results demonstrated that HSP90-WWP1/2-NEDD4L axis could regulate radiation-induced ferroptosis of HeLa cells.

**Fig. 6:**
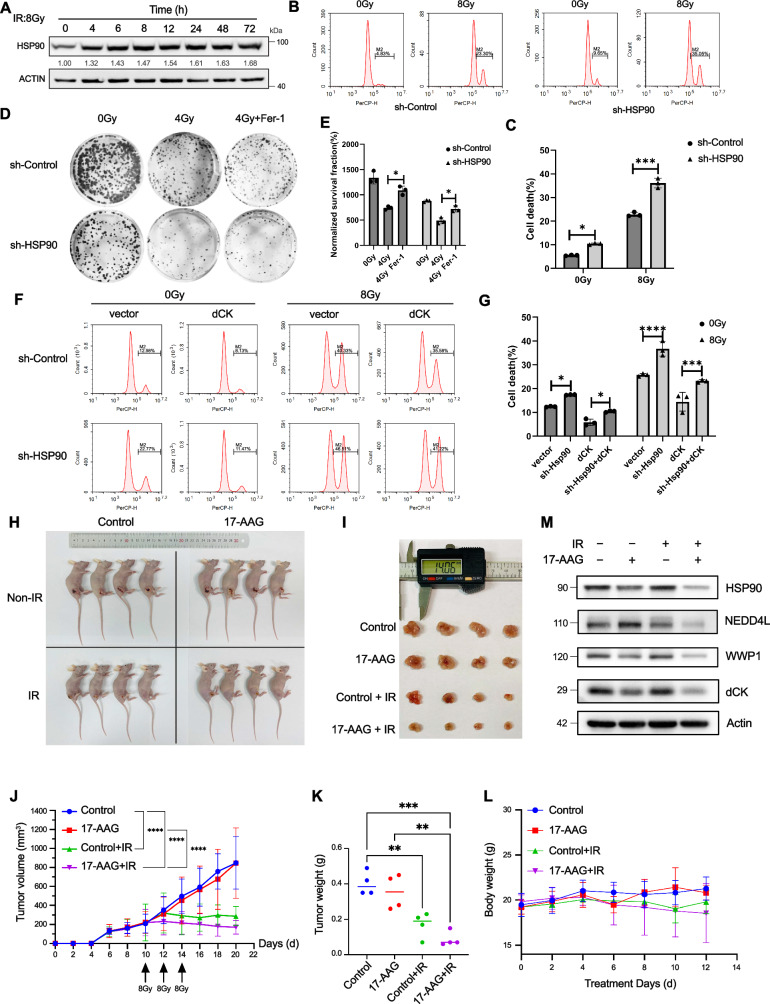
dCK could rescue HSP90 inhibitor-induced ferroptosis with or without IR in HeLa cells. **A** The degree of HSP90 expression in HeLa cells following 8 Gy of IR at various times. **B**, **C** The assessment of cell death in sh-Control and sh-HSP90 HeLa cells 48 h following an 8 Gy IR exposure. **D**, **E** The assay for colony formation in sh-Control and sh-HSP90 HeLa cells that underwent 2 h of pretreatment with 4 Gy IR or 20 μM ferrostatin-1 (fer-1). **F**, **G** Evaluation of cell death in sh-Control and sh-HSP90 HeLa cells exposed to 8 Gy of IR for 48 h, either with or without dCK expression. **H**–**L** Tumor volumes, tumor weights and body weights of mice were taken and measured after 17AAG (50 mg/kg) or IR (8 Gy×3) treatment. **M** Western blot analysis of animal tumour tissues. The error bars show the means ± SD from three independent repeats. *p* < 0.05 for *, *p* < 0.01 for **, and *p* < 0.001 for ***. Using a two-tailed unpaired student’s *t*-test, *P* values were determined.

We discovered that 8 Gy of IR boosted HSP90 protein expression in a time-dependent way (Fig. 6A). Furthermore, we discovered that HSP90 knockdown HeLa cells had higher rates of cell death (Fig. 6B, C). We carried out colony formation experiments using 4 Gy of IR with or without Fer-1 with the objective to elucidate the types of cell death brought on by HSP90 knockdown. The findings demonstrated that the ferroptosis pathways were the main mechanisms by which HSP90 knockdown caused cell death (Fig. 6D, E). It was noteworthy to note that following 4 Gy of IR treatment, there was a decrease in the proliferative capability of HeLa cells knocked down with HSP90. This indicated that HSP90 knockdown enhanced the radiosensitivity of HeLa cells, which could be restored by overexpressing dCK (Fig. 6F, G).To further confirm the role of HSP90 in vivo, we constructed a cervical cancer model in nude mice, which showed that the HSP90 inhibitor 17-AAG was able to reduce the tumour volume and tumour after radiation without any significant effect on the body weight of the mice(Fig. 6H–L), which further demonstrated the role of 17-AAG as a radiosensitizing drug for cervical cancer. Meanwhile, the regulatory role of 17-AAG for HSP90-WWP1-NEDD4L-dCK was also verified (Fig. 6M),

### High expression of HSP90 and dCK was associated with poor prognosis in CESC patients

**Fig. 7:**
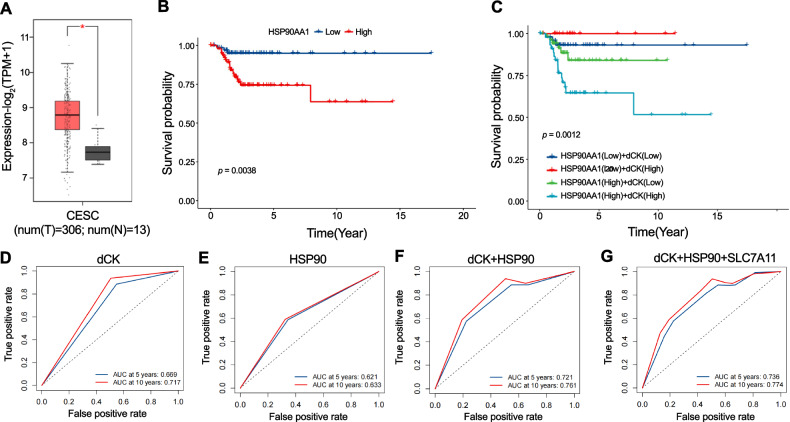
High expression of HSP90 and dCK was associated with poor prognosis in CESC patients. **A** HSP90AA1 comparison between normal tissues and CESC tumors. **B**, **C** Kaplan–Meier curves of RFS in cervical cancer according to the HSP90AA1, dCK and HSP90AA1 expression. **(D–G)** ROC curves for dCK, HSP90AA1, dCK+HSP90AA1, dCK+HSP90AA1 + SLC7A11, at the 5- and 10-year RFS. The error bars show the means ± SD from three independent repeats. *p* < 0.05 for *, *p* < 0.01 for **, and *p* < 0.001 for ***. Using a two-tailed unpaired student’s *t*-test, *P* values were determined.

By comparing tumor tissues to normal tissues, HSP90AA1 was increased (Fig. 7A). According to survival analysis, CESC patients with high expression of HSP90AA1 had considerably worse RFS (recurrence-free survival) than patients with low expression (Fig. 7B), indicating a strong negative correlation between high expression of HSP90AA1 and a bad prognosis in CESC patients. The expression levels of the two genes allowed us to further split the CESC patients into four groups. The survival analysis showed that the patients with high expression levels of both dCK and HSP90 had the worst prognosis (Fig. 7C). According to the ROC curves (Fig. 7D–G), the 5-year RFS AUC of dCK、HSP90、dCK+HSP90 and dCK+HSP90 + SLC7A11 was 0.669, 0.621,0.721 and 0,736, while the 10-year RFS AUC was 0.717, 0.633, 0.761 and 0.774. These results indicated that the combined genes had a much higher AUC than the individual genes.

## Discussion

One conventional method of treating cervical cancer is radiotherapy. However, the primary reason for treatment failure and a bad prognosis is the cancer cells’ resistance to radiation [16]. One crucial rate-limiting component of the nucleoside salvage pathway is deoxycytidine kinase (dCK) [17]. Deoxyribonucleotide triphosphates (dNTPs) are produced by dCK, which makes it a crucial component of DNA damage and repair [18, 19]. Clinical trials have employed dCK as an inhibitor of various chemotherapeutic medications [20–22]. In this study, dCK expression in cervical cancer was elevated by radiation. It was discovered that dCK knockdown exacerbated IR-induced cell death, primarily ferroptosis. Ferroptosis is a controlled cell death process caused by phospholipid peroxidation [23–26]. Mechanistically, dCK may partially activate the SLC7A11 promoter through the transcription factor ESR1. However, more research is needed to determine the precise mechanism by which ESR1 regulates SLC7A11.

Heat shock protein 90 (HSP90) is a chaperone that has undergone significant conservation and is widely distributed within the body [27, 28]. HSP90 inhibitors are currently being used in clinical trials to treat a variety of cancers, and they have shown some promise [29–31]. In our earlier work, we used mass spectroscopy to determine that HSP90 was a direct binding partner of dCK [32], and we confirmed it by performing Co-IP assays. In accordance to more research, HSP90 controlled the ubiquitin-proteasome pathway to control the protein stability of dCK. The primary mechanisms of protein degradation in eukaryotic cells are the ubiquitin-proteasome and autophagy-lysosome pathways, which control numerous cellular functions and maintain a normal metabolic balance. NEDD4L was found to be an E3 ubiquitin-ligase of dCK in this investigation. On the other hand, overexpression of HSP90 shortened the half-life of NEDD4L protein. We investigated the underlying mechanisms because of the intriguing discovery. An article describes the regulatory relationship between de-ubiquitinase USP44 and ubiquitinase TRIM25 [33], which guided us to search for another ubiquitinase of NEDD4L, and finally selected WWP1/2. We found HSP90 could directly bind to WWP1/2, thus contribute to NEDD4L protein degradation. This result unveils a previously unidentified mechanism by which HSP90 encourages dCK degradation in HeLa cells by ubiquitinating NEDD4L via WWP1/2.

**Fig. 8:**
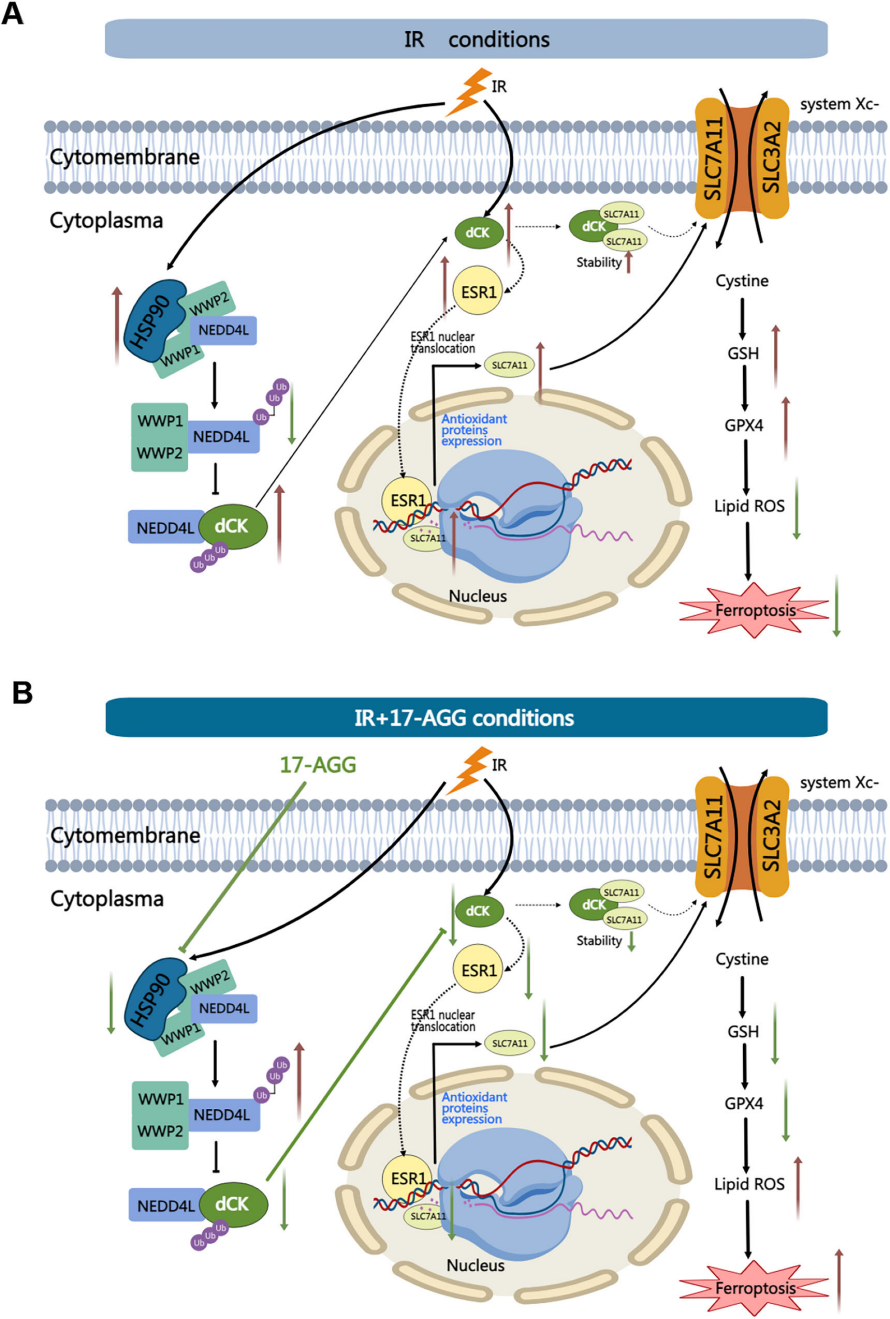
HSP90-WWP1/WWP2-NEDD4L-dCK-ESR1-SLC7A11 axis regulated IR induced ferroptosis axis. **A** Inhibition of cellular ferroptosis by the HSP90-WWP1/WWP2-NEDD4L-dCK-ESR1-SLC7A11 axis under normal radiation conditions. **B** Promotion of cellular ferroptosis by the HSP90-WWP1/WWP2-NEDD4L-dCK-ESR1-SLC7A11 axis after using HSP90 inhibitor (17-AAG) under radiation conditions.

Although dCK has been reported as a prognostic indicator in gastric cancer [34], liver cancer [35] and meningioma [36], the correlation between survival and the joint expression of dCK and HSP90 in cervical cancer remains elusive. Here, our data showed that the worst prognosis was seen in CESC patients who had high expression of both dCK and HSP90. Potential treatment targets for CESC are offered by these findings. In conclusion, HSP90 stabilizes dCK protein and inhibits NEDD4L under IR conditions by enlisting the ubiquitin ligases WWP1/2. HeLa cells are resistant to ferroptosis and apoptosis because dCK enhanced SLC7A11 transciption via ESR1. HSP90 will be efficiently inhibited while IR is combined with 17-AAG treatment, which supports the HSP90-WWP1/2-NEDD4L-dCK axis’ antitumor effect (Fig. 8). As a result of our research, we have concluded that dCK might be a new therapeutic target for increasing the radiosensitivity of HeLa cells and that HSP90 inhibitors might help to increase the effectiveness of radiation therapy for cervical cancer.

## Materials and methods

### Reagents and antibodies

dCK (ab186128), transferrin (ab8241) and Fas (ab8009) were purchased from Abcam (Cambridge, MA, USA); SLC7A11 (#12691S), CD71 (#13113S), Bcl-2 (#3498S), BAX (#2772), TFAM (#7495S), PGC1α (#2178S), DRP1 (#5391S), HSP90AA1 (#4874) and NEDD4L (#4001) was purchased from Cell Signaling Technology (Danvers, MA, USA).

### Cell culture

The human embryonic kidney (HEK) 293T cell line and the human-source CESC HeLa cell line were obtained from the Chinese Academy of Sciences’ cell bank in Beijing, China, and were routinely examined for the presence of mycoplasma. Every cell was regularly cultivated at 37 °C in a humidified environment with 5% CO_2_ in Dulbecco’s modified Eagle’s medium (DMEM) (Gibco, USA), which contained 10% fetal bovine serum (Gibco, USA) and 1% penicillin/streptomycin (Cat. No. 10378016, Life Technologies).

### Irradiation

Radiation was delivered using an X-RAY generator (X-RAD 320 ix, Precision X-ray Inc., North Branford, CT, USA). Conditions of the radiation: 320 kV, 12.5 mA, filter 1, 50 cm (standard deviation) distance, 300 cGy/min dose rate, 8 Gy single dose in cells.

### Experimental animals

BALB/c-nude mice were purchased from Jiangsu Jicui Pharmachem Biological Co. Five-week-old male nude mice weighing 20–22 g were kept without restriction of food and water intake. They were kept at room temperature (22 ± 2)°C, relative humidity 50–60%, and alternating light and dark conditions for 12 h. A suspension of 2 × 10^6^ Hela cells was injected into the right leg of nude mice subcutaneously, The experimental animals were divided into control (*n* = 4), 17-AAG (*n* = 4), control+IR (*n* = 4) and 17-AAG + IR (*n* = 4) groups. The tumour changes of nude mice were observed every 3 days after subcutaneous tumour formation, IR and 17-AAG treatment were given when the tumour volume reached 100 mm^3^, and a lead mould was used for irradiation to protect the rest of the normal tissues outside the tumour, and when the tumour was close to 1.5 cm in length and diameter, the nude mice were put to death, the ruffed mice were photographed, and the tumour was peeled off, and weighed.

### Colony formation assay

The cells were cultivated in DMEM (Invitrogen) with 10% FBS (Gibco) at 37 °C and 5% CO_2_ in 6-well plates. The cells were exposed to 4 Gy of radiation 24 h later. Following a 14-day incubation period, the colonies were stained for 15 minutes at room temperature with 0.2% crystal violet (Solarbio, Beijing, China) and fixed for 30 min with 4% paraformaldehyde (Solarbio, Beijing, China). Colonies with at least 50 cells per dish were tallied.

### Flow cytometric analysis

The cell pellet was stained with 200 µL of PBS containing 0.04% of trypan blue, and cell death was then measured by flow cytometry (ACEA NovoCyte 2040R, USA). Lipid peroxidation was assessed by flow cytometry with BODIPY™ 581/591C11 (ThermoFisher Scientific, USA) following the manufacturer’s instruction.

### Lentiviral production

Lentiviral short hairpin RNA (shRNA) vector targeting HSP90 and dCK was constructed according to the protocol of pLKO.1-blasticidin vector (Addgene, Cambridge, MA, USA).

### Western blot and immunoprecipitation (IP)

Cells were lysed in RIPA Lysis Buffer for Western blot analysis. Following the manufacturer’s instructions, the supernatant was gathered, and the protein content was ascertained using the BCA Protein Assay Kit (ThermoFisher, USA). After being separated by 10% or 12% SDS-PAGE, equal amounts of total protein (20 µg) were transferred to an equilibrated PVDF membrane (Millipore, USA). incubation with the primary antibody and an anti-mouse or anti-rabbit secondary antibody (1:10,000) conjugated with peroxidase. For IP, Cells were lysed in 1% NP-40 lysis buffer (137 mM NaCl, 10 mM NaF, 50 mM Tris-HCl (pH 7.6), 1 mM EDTA, 0.1 mM Na_3_VO_4_, 10% glycerol, 1% NP-40, and 1 mM PMSF). Cell lysates were either incubated for 3 h at 4 °C with the appropriate antibody and then for 3 h or overnight at 4 °C with protein A/G-Sepharose beads. Alternatively, cell lysates were incubated with anti-FLAG beads. The protein-antibody complexes were then resolved by SDS-PAGE and Western blot after being washed three times at 4 °C with cold lysis buffer and eluted with SDS loading buffer by boiling for 10 minutes.

### Immunofluorescence co-localization assay

A total of 48 h after transfection, cells were separated into plates, and slides were added before being divided. The cells were collected when they had completely adhered to the coverslip, fixed with 4% paraformaldehyde for 20 min, and then subjected to 0.1% TritonX-100 treatment for 15 min. After blocking the cells with 10% normal non-immune goat serum for 1 h, the cells were treated with the appropriate primary antibodies overnight. The next day, cells were incubated with the corresponding secondary fluorescent antibodies for 1 h and then washed with PBS-tween for 5 min × 3 times. The slides were dried at room temperature avoiding light, then antifade solution was added.

### Luciferase reporter assays

The pGL3-Basic vector was inserted with reporter plasmids of the SLC7A11 promoter double luciferase that were synthesized from Generalbiol (Anhui, China). Cells with PCDNA3.1-Flag ESR1 and PCDNA3.1-Flag dCK were co-transfected with the pGL3-Basic SLC7A11 promoter and pGL3-Basic vector, respectively. Using a Centro LB 960 Luminometer (Berthold Technologies, Germany) to measure luciferase activity 48 h after transfection, Renilla luciferase activity was utilized as a standard control.

### RT-qPCR

Total RNA was extracted from cells with Trizol solution (TaKaRa, Dalian, China). PrimeScriptTM RT Master Mix (TaKaRa, Dalian, China) was used for reverse transcription. qRT-PCR was carried out on a QuantStudio real-time PCR instrument (ThermoFisher Scientific, USA) using SYBR Premix Ex Taq II (TaKaRa). The performed as the following thermal conditions: 95 °C for 30 s followed by 40 cycles of 95 °C for 5 s and 60 °C for 30 s. qPCR was performed on QuantStudio 3 (Applied Biosystems, United States), and the expression of target genes was normalized to that of GAPDH, all the primers listed in Supplementary Table 1.

### Cell viability assays

Cell Counting Kit-8 (CCK-8, Dojindo Laboratories, Japan) was used to assess cell viability in accordance with the manufacturer’s instructions. The cells were treated with drugs after being seeded in 96-well plates with 2 × 10^3^ cells per well. Each well was supplemented with CCK-8, and the cells were cultured for 4 h. OD values were measured with a microplate reader at 450 nm. The proliferation rate of the cells was calculated by the following formula: cell viability = (OD experimental group − OD blank)/(OD control group − OD blank) × 100%.

### Statistical analysis

The data was displayed as mean ± SD. With the aid of GraphPad Prism 8 Software (San Diego, CA, USA), statistical analyses were carried out. An ANOVA was used for the experiments. *P* < 0.05 was the threshold for statistical significance, and all *P* values were two-sided. R software (version 4.1.2, http://www.r-project.org/) was used for the predictive analysis and visualization. The R packages “survival”, “survminer”, “survival ROC”, and “rms” are utilized.

## Supplementary information

Supplementary figure and legends

Original Data

## Data Availability

All data generated or analyzed during this study are included in this published article [and its supplementary information files].

## References

[1] Arbyn M, Weiderpass E, Bruni L, de Sanjosé S, Saraiya M, Ferlay J, et al. Estimates of incidence and mortality of cervical cancer in 2018: a worldwide analysis. Lancet Glob Health. 2020;8:e191–e203. 31812369 10.1016/S2214-109X(19)30482-6 PMC7025157

[2] Poflee SV, Bhatia JK. Cervical cytology: radiation and other therapy effects. Cytojournal. 2022;19:32. 35673693 10.25259/CMAS_03_12_2021 PMC9168396

[3] Tsikouras P, et al. Cervical cancer: screening, diagnosis and staging. J BUON. 2016;21:320–5. 27273940

[4] Tsikouras P, Zervoudis S, Manav B, Tomara E, Iatrakis G, Romanidis C, et al. Molecular definitions of cell death subroutines: recommendations of the Nomenclature Committee on Cell Death 2012. Cell Death Differ. 2012;19:107–20. 21760595 10.1038/cdd.2011.96 PMC3252826

[5] Penaloza C, Lin L, Lockshin RA, Zakeri Z. Cell death in development: shaping the embryo. Histochem Cell Biol. 2006;126:149–58. 16816938 10.1007/s00418-006-0214-1

[6] Baidoo K, Yong K, Brechbiel M. Molecular pathways: targeted α-particle radiation therapy. Clin Cancer Res. 2013;19:530–7. 23230321 10.1158/1078-0432.CCR-12-0298 PMC3563752

[7] Hanahan D, Weinberg R. Hallmarks of cancer: the next generation. Cell. 2011;144:646–74. 21376230 10.1016/j.cell.2011.02.013

[8] Conrad M, Angeli JP, Vandenabeele P, Stockwell BR. Regulated necrosis: disease relevance and therapeutic opportunities. Nat Rev Drug Discov. 2016;15:348–66. 26775689 10.1038/nrd.2015.6 PMC6531857

[9] Arnér E, Eriksson S. Mammalian deoxyribonucleoside kinases. Pharmacol Ther. 1995;67:155–86. 7494863 10.1016/0163-7258(95)00015-9

[10] Lee MW, Parker WB, Xu B. New insights into the synergism of nucleoside analogs with radiotherapy. Radiat Oncol. 2013;8:223. 24066967 10.1186/1748-717X-8-223 PMC3851323

[11] Kerr M, Scott HE, Groselj B, Stratford MR, Karaszi K, Sharma NL, et al. Deoxycytidine kinase expression underpins response to gemcitabine in bladder cancer. Clin Cancer Res. 2014;20:5435–45. 25224279 10.1158/1078-0432.CCR-14-0542 PMC4216732

[12] Schulte TW, Neckers LM. The benzoquinone ansamycin 17-allylamino-17-demethoxygeldanamycin binds to HSP90 and shares important biologic activities with geldanamycin. Cancer Chemother Pharmacol. 1998;42:273–9. 9744771 10.1007/s002800050817

[13] Sato H, Nomura S, Maebara K, Sato K, Tamba M, Bannai S. Transcriptional control of cystine/glutamate transporter gene by amino acid deprivation. Biochem Biophys Res Commun. 2004;325:109–16. 15522208 10.1016/j.bbrc.2004.10.009

[14] Liu R, Liu L, Bian Y, Zhang S, Wang Y, Chen H, et al. The dual regulation effects of ESR1/NEDD4L on SLC7A11 in breast cancer under ionizing radiation. Front Cell Dev Biol. 2021;9:772380. 35252218 10.3389/fcell.2021.772380 PMC8888677

[15] Bunimovich YL, Nair-Gill E, Riedinger M, McCracken MN, Cheng D, McLaughlin J, et al. Deoxycytidine kinase augments ATM-Mediated DNA repair and contributes to radiation resistance. PLoS ONE. 2014;9:e104125. 25101980 10.1371/journal.pone.0104125 PMC4125169

[16] Conrad M, Sato H. The oxidative stress-inducible cystine/glutamate antiporter, system x (c) (-) : cystine supplier and beyond. Amino Acids. 2012;42:231–46. 21409388 10.1007/s00726-011-0867-5

[17] Dixon SJ, Lemberg KM, Lamprecht MR, Skouta R, Zaitsev EM, Gleason CE, et al. Ferroptosis: an iron-dependent form of nonapoptotic cell death. Cell. 2012;149:1060–72. 22632970 10.1016/j.cell.2012.03.042 PMC3367386

[18] Sasaki H, Sato H, Kuriyama-Matsumura K, Sato K, Maebara K, Wang H, et al. Electrophile response element-mediated induction of the cystine/glutamate exchange transporter gene expression. J Biol Chem. 2002;277:44765–71. 12235164 10.1074/jbc.M208704200

[19] Hu Q, Qin Y, Xiang J, Liu W, Xu W, Sun Q, et al. dCK negatively regulates the NRF2/ARE axis and ROS production in pancreatic cancer. Cell Prolif. 2018;51:e12456. 29701272 10.1111/cpr.12456 PMC6528851

[20] Sarto C, Binz PA, Mocarelli P. Heat shock proteins in human cancer. Electrophoresis. 2000;21:1218–26. 10786894 10.1002/(SICI)1522-2683(20000401)21:6<1218::AID-ELPS1218>3.0.CO;2-H

[21] Li L, Gong Y, Xu K, Chen W, Xia J, Cheng Z, et al. ZBTB28 induces autophagy by regulation of FIP200 and Bcl-XL facilitating cervical cancer cell apoptosis. J Exp Clin Cancer Res. 2021;40:150. 33931087 10.1186/s13046-021-01948-0 PMC8086320

[22] Liu HM, Ji F, Lu Y, SY Chen. MiR-499b-5p inhibits cervical cancer cell proliferation and induces apoptosis by targeting the Notch1 signaling pathway. Eur Rev Med Pharmacol Sci. 2021;25:6220–31. 34730202 10.26355/eurrev_202110_26992

[23] Meng Y, Sun H, Li Y, Zhao S, Su J, Zeng F, et al. Targeting ferroptosis by ubiquitin system enzymes: a potential therapeutic strategy in cancer. Int J Biol Sci. 2022;18:5475–88. 36147464 10.7150/ijbs.73790 PMC9461661

[24] Lei G, Zhang Y, Koppula P, Liu X, Zhang J, Lin SH, et al. The role of ferroptosis in ionizing radiation-induced cell death and tumor suppression. Cell Res. 2020;30:146–62. 31949285 10.1038/s41422-019-0263-3 PMC7015061

[25] Wang C, Zeng J, Li LJ, Xue M, He SL. Cdc25A inhibits autophagy-mediated ferroptosis by upregulating ErbB2 through PKM2 dephosphorylation in cervical cancer cells. Cell Death Dis. 2021;12:1055. 34743185 10.1038/s41419-021-04342-y PMC8572225

[26] Zhao MY, Liu P, Sun C, Pei LJ, Huang YG. Propofol augments paclitaxel-induced cervical cancer cell ferroptosis in vitro. Front Pharmacol. 2022;13:816432. 35517791 10.3389/fphar.2022.816432 PMC9065257

[27] Arkhypov I, Özbay Kurt FG, Bitsch R, Novak D, Petrova V, Lasser S. et al. HSP90α induces immunosuppressive myeloid cells in melanoma via TLR4 signaling. J Immunother Cancer. 2022;10:e005551. 36113897 10.1136/jitc-2022-005551 PMC9486388

[28] Zhang K, Wang M, Li Y, Li C, Tang S, Qu X, et al. The PERK-EIF2α-ATF4 signaling branch regulates osteoblast differentiation and proliferation by PTH. Am J Physiol Endocrinol Metab. 2019;316:E590–e604. 30668150 10.1152/ajpendo.00371.2018

[29] Kawazoe A, Itahashi K, Yamamoto N, Kotani D, Kuboki Y, Taniguchi H, et al. TAS-116 (Pimitespib), an oral HSP90 inhibitor, in combination with nivolumab in patients with colorectal cancer and other solid tumors: an open-label, dose-finding, and expansion phase Ib trial (EPOC1704). Clin Cancer Res. 2021;27:6709–15. 34593531 10.1158/1078-0432.CCR-21-1929

[30] Cercek A, Shia J, Gollub M, Chou JF, Capanu M, Raasch P, et al. Ganetespib, a novel Hsp90 inhibitor in patients with KRAS mutated and wild type, refractory metastatic colorectal cancer. Clin Colorectal Cancer. 2014;13:207–12. 25444464 10.1016/j.clcc.2014.09.001 PMC5489410

[31] Slovin S, Hussain S, Saad F, Garcia J, Picus J, Ferraldeschi R, et al. Pharmacodynamic and Clinical Results from a Phase I/II Study of the HSP90 Inhibitor Onalespib in Combination with Abiraterone Acetate in Prostate Cancer. Clin Cancer Res. 2019;25:4624–33. 31113841 10.1158/1078-0432.CCR-18-3212 PMC9081826

[32] Yang C, Lee M, Hao J, Cui X, Guo X, Smal C, et al. Deoxycytidine kinase regulates the G2/M checkpoint through interaction with cyclin-dependent kinase 1 in response to DNA damage. Nucleic Acids Res. 2012;40:9621–32. 22850745 10.1093/nar/gks707 PMC3479177

[33] Chen Y, Zhao Y, Yang X, Ren X, Huang S, Gong S, et al. USP44 regulates irradiation-induced DNA double-strand break repair and suppresses tumorigenesis in nasopharyngeal carcinoma. Nat Commun. 2022;13:501. 35079021 10.1038/s41467-022-28158-2 PMC8789930

[34] Wen F, Huang J, Lu X, Huang W, Wang Y, Bai Y, et al. Identification and prognostic value of metabolism-related genes in gastric cancer. Aging. 2020;12:17647–61. 32920549 10.18632/aging.103838 PMC7521523

[35] He S, Qiao J, Wang L, Yu L. A novel immune-related gene signature predicts the prognosis of hepatocellular carcinoma. Front Oncol. 2022;12:955192. 36185203 10.3389/fonc.2022.955192 PMC9520462

[36] Yamamoto M, Sanomachi T, Suzuki S, Uchida H, Yonezawa H, Higa N, et al. Roles for hENT1 and dCK in gemcitabine sensitivity and malignancy of meningioma. Neuro Oncol. 2021;23:945–54. 33556172 10.1093/neuonc/noab015 PMC8168817

