# Supplementary material for: HSP90 regulates dCK stability and inhibits ionizing radiation-induced ferroptosis in cervical cancer cells
Source: Cell Death Discov. 2025 Apr 22;11:191. doi: 10.1038/s41420-025-02388-x (PMC12015294; doi:10.1038/s41420-025-02388-x)
Supplement: Supplementary file 1 — Supplementary figure and legends [file 41420_2025_2388_MOESM1_ESM.pdf]

Supplementary figure S1

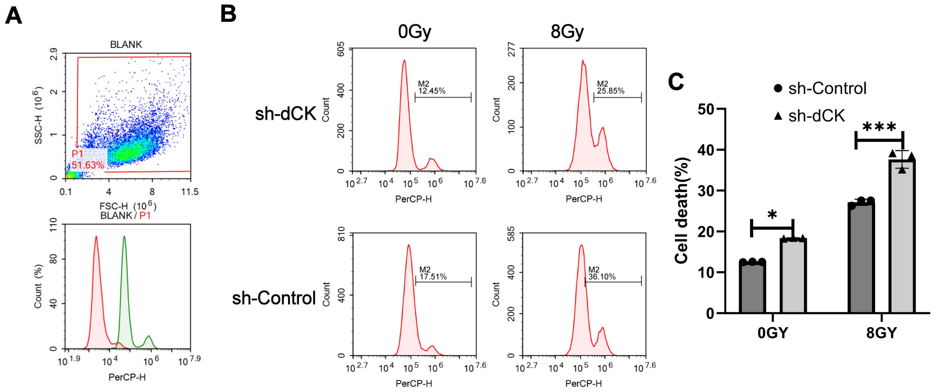

Supplementary figure S2

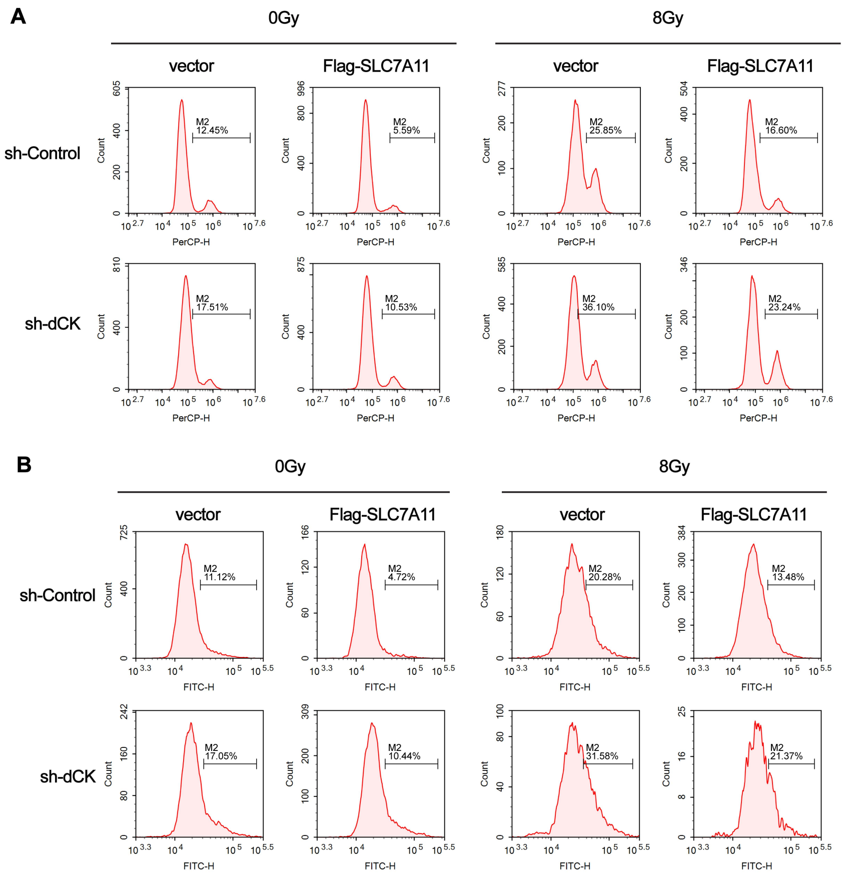

Supplementary figure S3

A

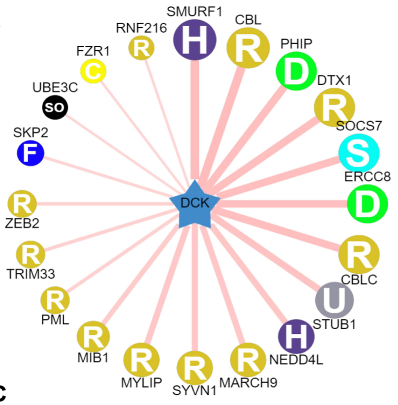

B

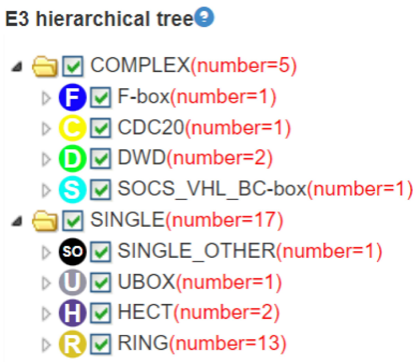

C

E3	E3GENE	SUB	SUBGENE	HOMO	PFAM	GO	NET	MOTIF	SCORE
P22681	CBL	P27707	DCK	1	1	4.05	1	1.06	0.653
Q9HCE7	SMURF1	P27707	DCK	1	1	4.05	1	1.06	0.653
O14512	SOCS7	P27707	DCK	1	1	4.05	1	1	0.647
Q9ULV8	CBLC	P27707	DCK	1	1	4.05	1	1	0.647
Q13216	ERCC8	P27707	DCK	1	1	4.05	1	1	0.647
Q86Y01	DTX1	P27707	DCK	1	1	4.05	1	1	0.647
Q8WWQ0	PHIP	P27707	DCK	1	1	4.05	1	1	0.647
Q9UNE7	STUB1	P27707	DCK	1	1	3.77	1	1	0.64
Q96PU5	NEDD4L	P27707	DCK	1	1	1.27	1	2.8	0.634
Q8WY64	MYLIP	P27707	DCK	1	1	1.25	1	2.8	0.633

D

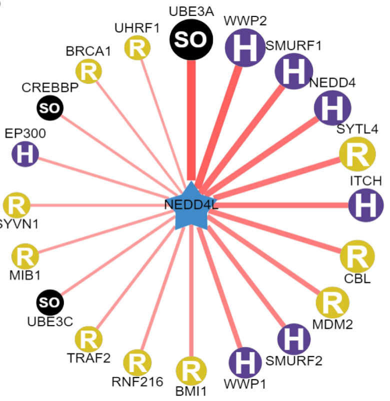

E

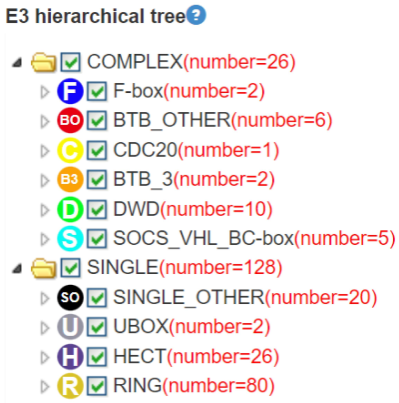

F

E3	E3GENE	SUB	SUBGENE	HOMO	PFAM	GO	NET	MOTIF	SCORE
Q05086	UBE3A	Q96PU5	NEDD4L	1	3.17	2.93	2.17	2.8	0.843
O00308	WWP2	Q96PU5	NEDD4L	1	3.17	2.93	1.98	2.12	0.821
Q9HCE7	SMURF1	Q96PU5	NEDD4L	1	3.17	4.05	2.39	1.06	0.802
P46934	NEDD4	Q96PU5	NEDD4L	1	3.17	4.43	2.26	1	0.793
Q96C24	SYTL4	Q96PU5	NEDD4L	1	3.17	4.43	1.44	1	0.787
Q96J02	ITCH	Q96PU5	NEDD4L	1	3.17	2.93	2.17	1.06	0.779
P22681	CBL	Q96PU5	NEDD4L	1	3.18	2.93	1.99	1.06	0.773
Q00987	MDM2	Q96PU5	NEDD4L	1	1	2.93	1.77	2.8	0.762
Q9HAU4	SMURF2	Q96PU5	NEDD4L	1	3.17	2.33	2.17	1.06	0.761
Q9H0M0	WWP1	Q96PU5	NEDD4L	1	3.17	2.93	1.7	1.06	0.76

Supplementary figure S4

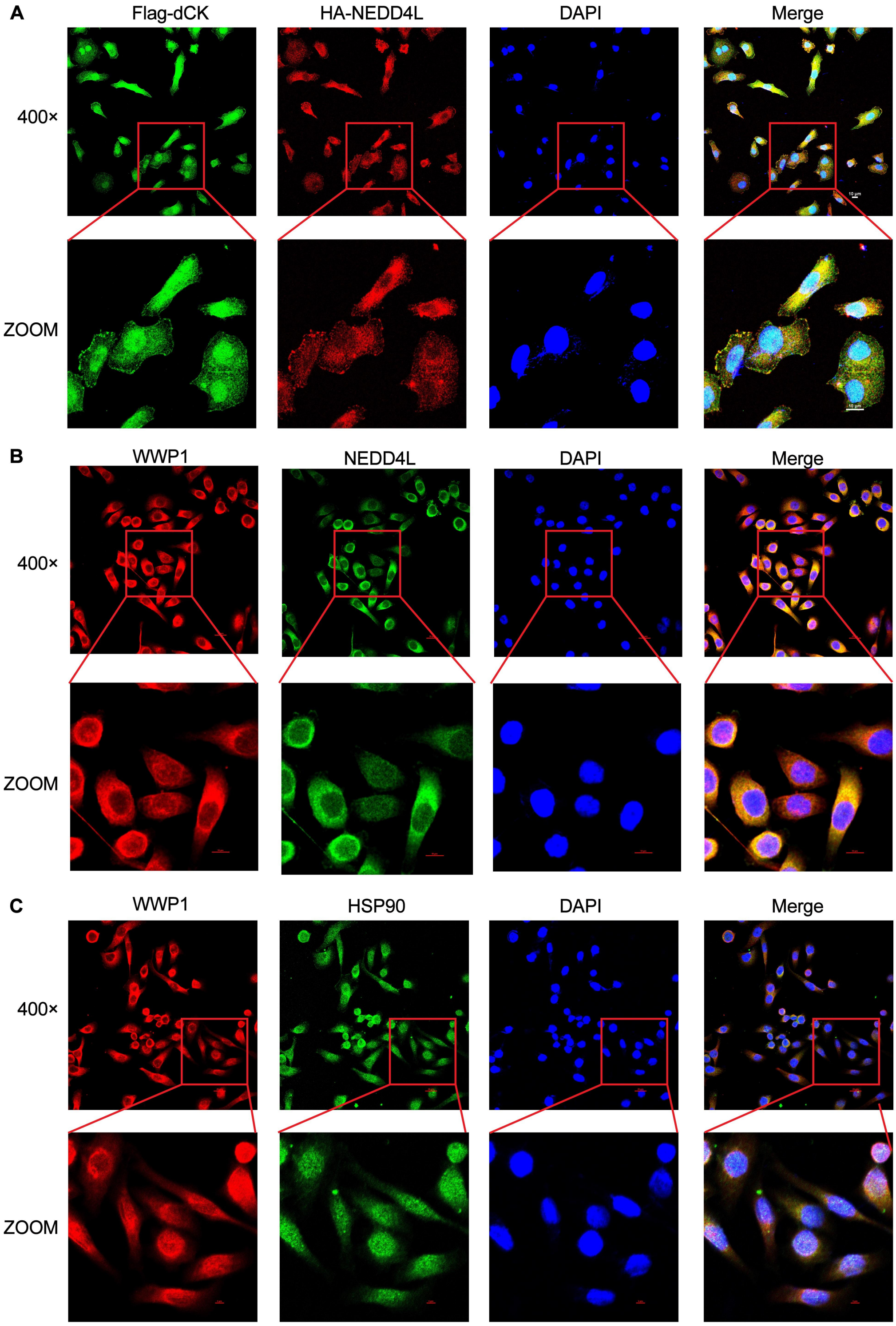

Supplementary legends

Supplymentary Figure S1.

(A-C) Evaluation of cell death in sh-Control and sh-dCK HeLa cells 48 hours following an 8 Gy IR exposure. Trypan blue staining of the identified cells' relative levels of cell death is displayed in a bar graph.The error bars represent means±SD based on three independent repeats. * Denotes non-significance; **, $p < 0.01$; ***, $p < 0.001$. Using a two-tailed unpaired student's t-test, P values were determined.

Supplymentary Figure S2.

(A)Evaluation of cell death in SLC7A11 expressing sh-Contorl and dCK knockdown HeLa cells at 48h after exposure to 8 Gy of IR.(B)Lipid peroxidation assessment of SLC7A11 expressing sh-Contorl and dCK knockdown HeLa cells at 48h after exposure to 8 Gy of IR. .

Supplymentary Figure S3.

(A-B)The E3 ubiquitination enzymes are predicted for dCK on the UbiBrowser website.(C)The top 10 gene information for the E3 ligase score for dCK. (D-E)The E3 ubiquitination enzymes are predicted for NEDD4L on the UbiBrowser website.(F)The top 10 gene information for the E3 ligase score for NEDD4L.

Supplymentary Figure S4.

The expression levels of HSP90 and WWP1、WWP1 and NEDD4L、NEDD4L and dCK in Hela cells were detected by immunofluorescence co-localisation assay.

Supplementary Table 1

Primer

GAPDH

Forward：5’-CCATGGGTGGAATCATATTGGA-3’

Reverse：5’-TCAACGGATTTGGTCGTATTGG-3’

dCK

Forward：5’-GCAGCCTGCTATAAAGTTAAA-3’

Reverse：5’-CCTTGAATTGGATGGAATCAT-3’

ESR1

Forward：5’-GCTTACTGACCAACCTGGCAGA-3’

Reverse：5’-GGATCTCTAGCCAGGCACATTC-3’

SLC7A11

Forward：5’-GCGTGGGCATGTCTCTGAC-3’

Reverse：5’-GCTGGTAATGGACCAAAGACTTC-3’

PTGS2

Forward：5’-TAGGATTCAGGGCTTTCACTGGCT-3’

Reverse：5’-TGTCAGCCGACAATGAGATGTGGA-3’

GPX4

Forward：5’-GAGGCAAGACCGAAGTAAACTAC-3’

Reverse：5’-CCGAACTGGTTACACGGGAA-3’
