# Supplementary material for: HSP90 regulates dCK stability and inhibits ionizing radiation-induced ferroptosis in cervical cancer cells
Source: Cell Death Discov. 2025 Apr 22;11:191. doi: 10.1038/s41420-025-02388-x (PMC12015294; doi:10.1038/s41420-025-02388-x)

Figure 1C

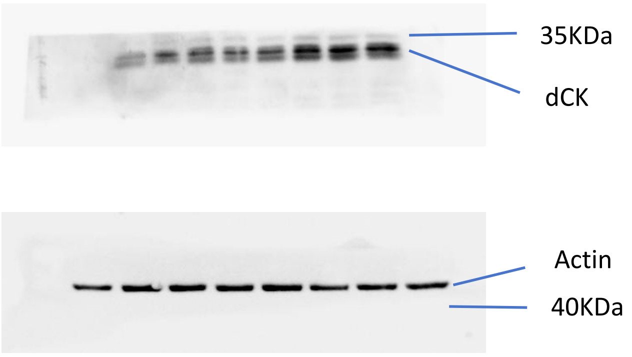

Figure 1D

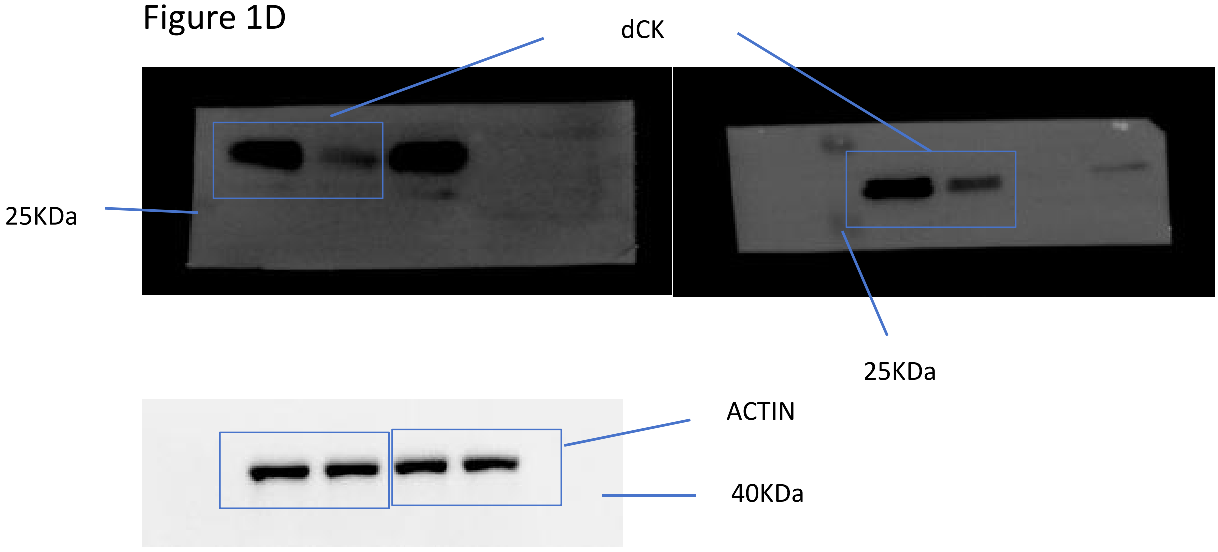

Figure 3A

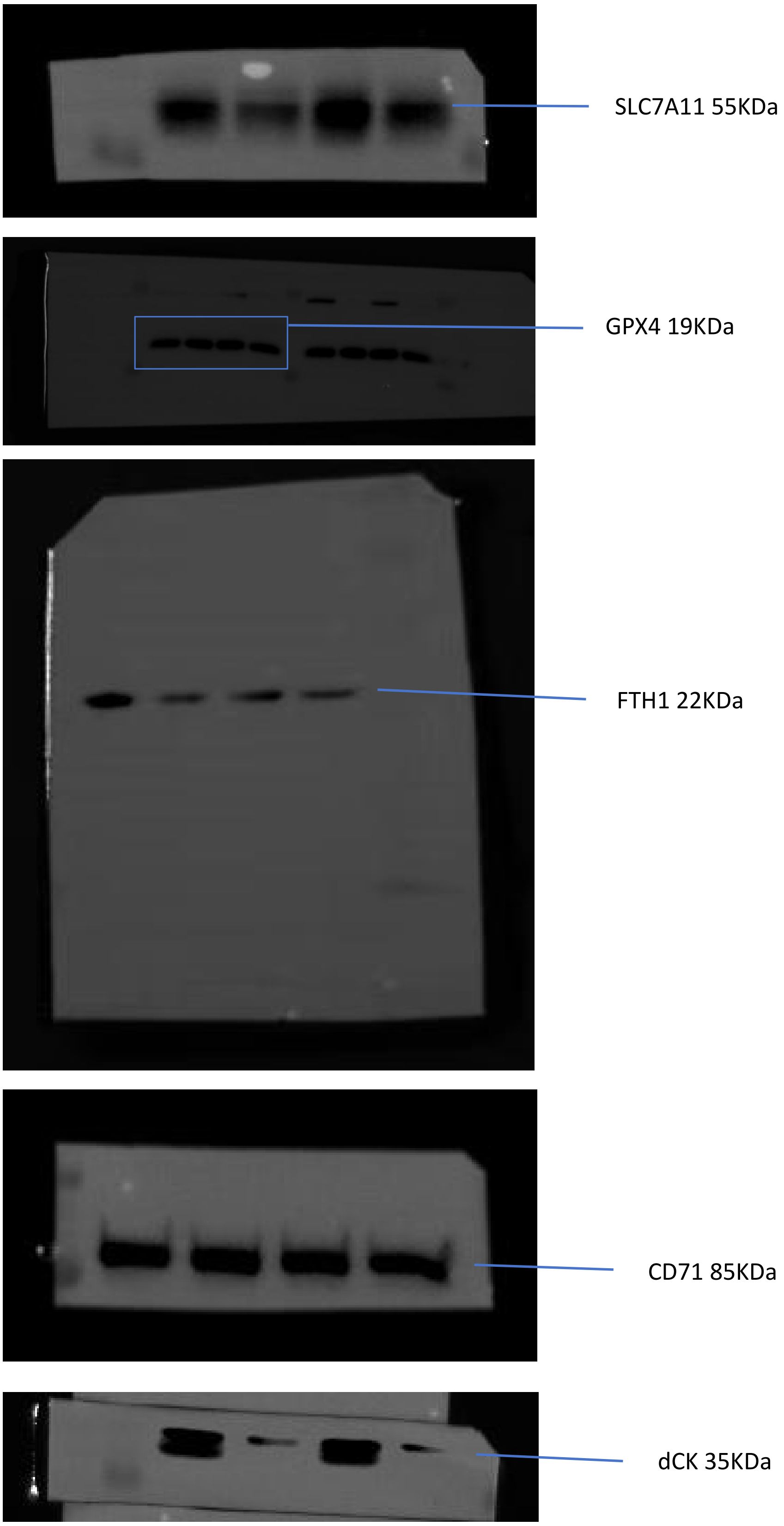

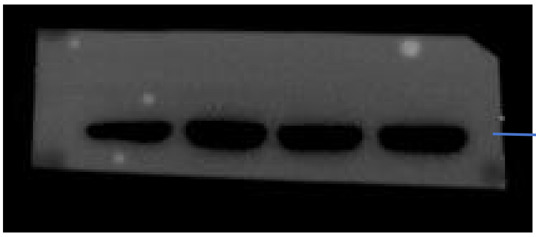

ACTIN 42KDa

Figure 3D

Flag-dCK

IP:Flag

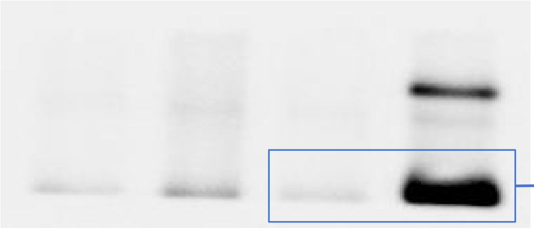

SLC7A11 55KDa

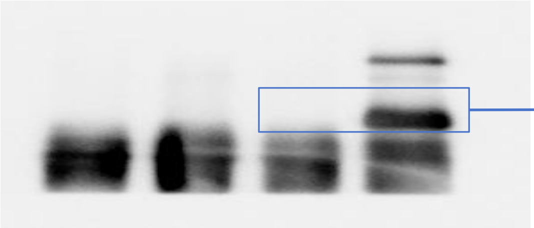

Flag 26KDa

Input

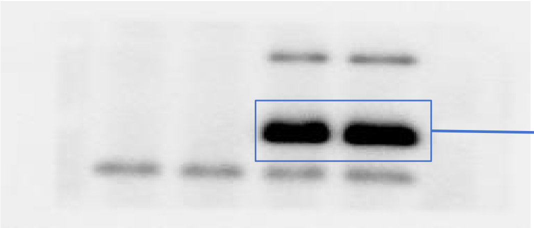

SLC7A11 55KDa

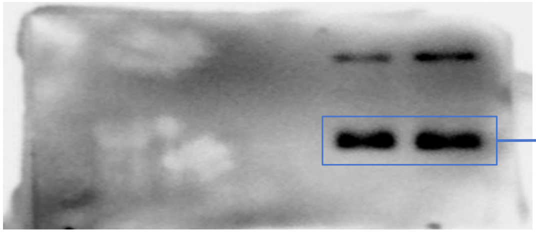

Flag 26KDa

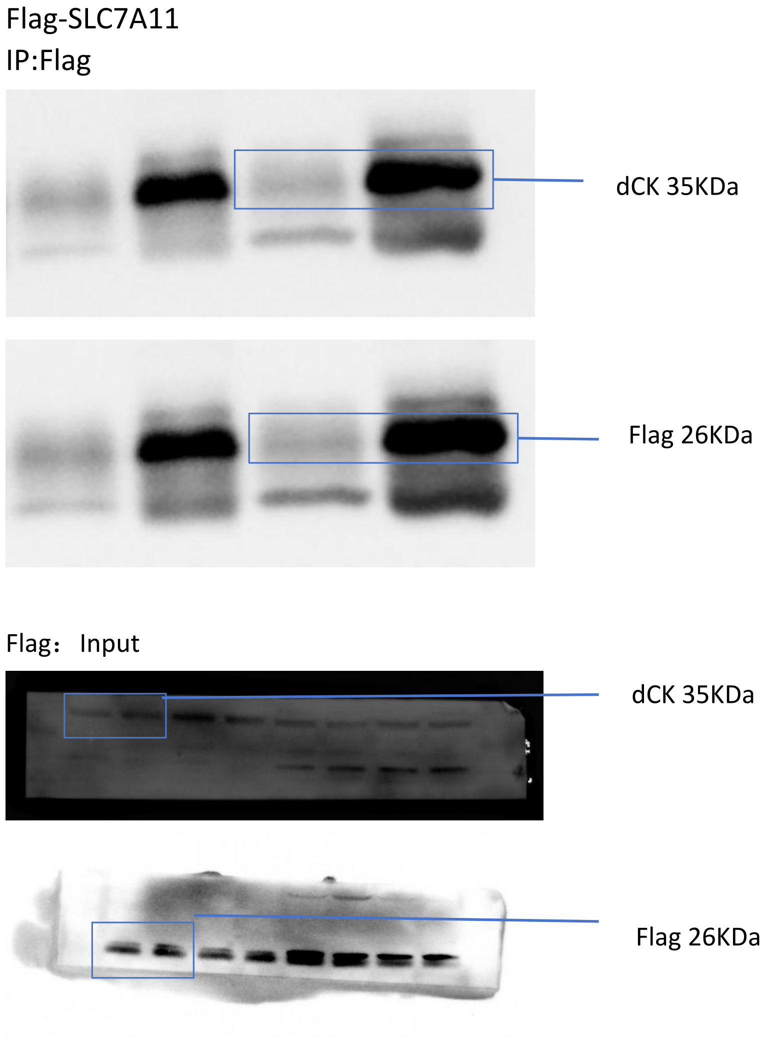
Flag-SLC7A11
IP:Flag
dCK 35KDa
Flag 26KDa
Flag：Input
dCK 35KDa
Flag 26KDa

Figure 3E

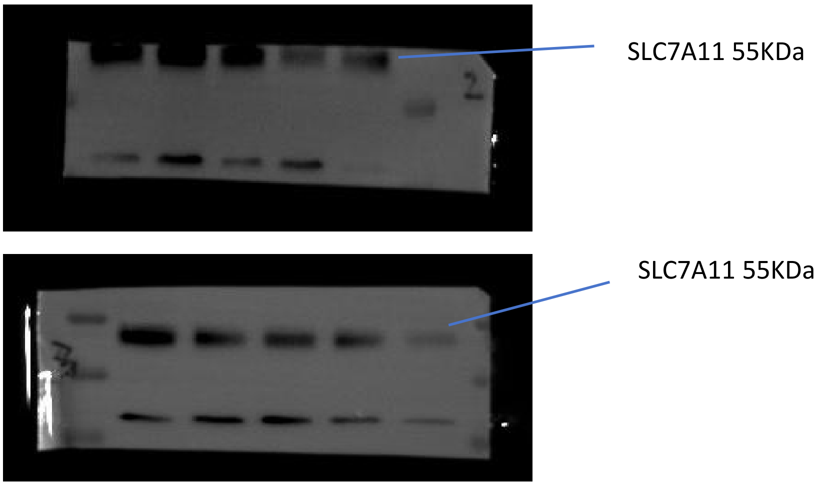
SLC7A11 55KDa
SLC7A11 55KDa

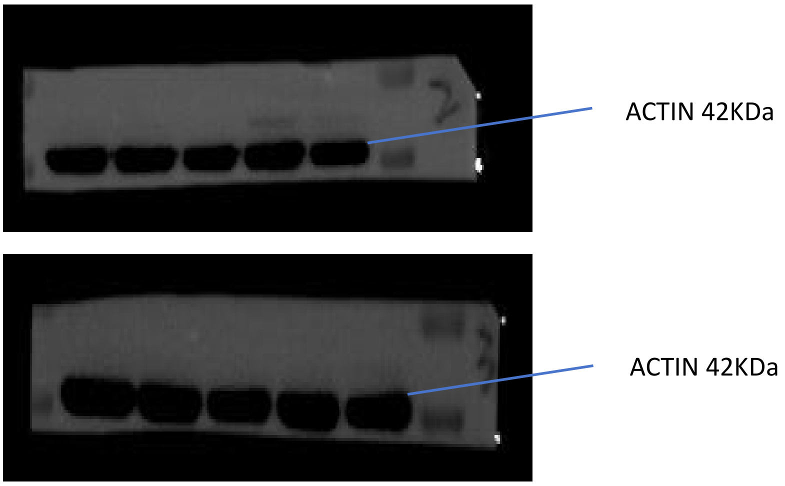

Figure 4B

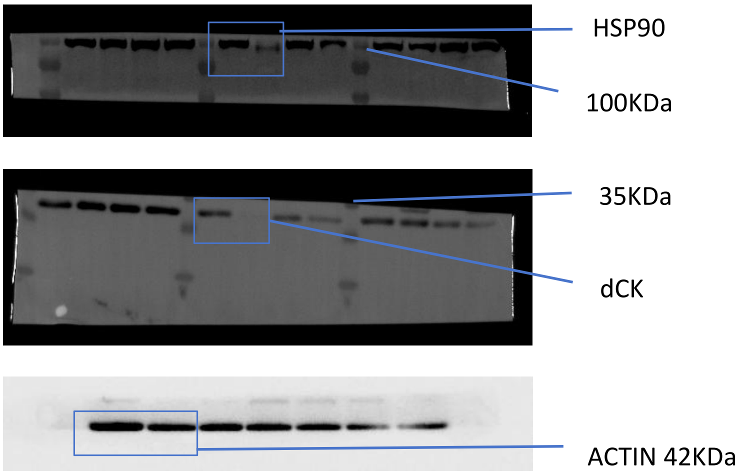

Figure 4C

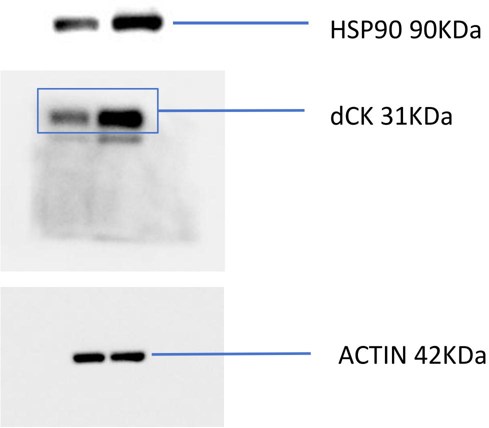

Figure 4D

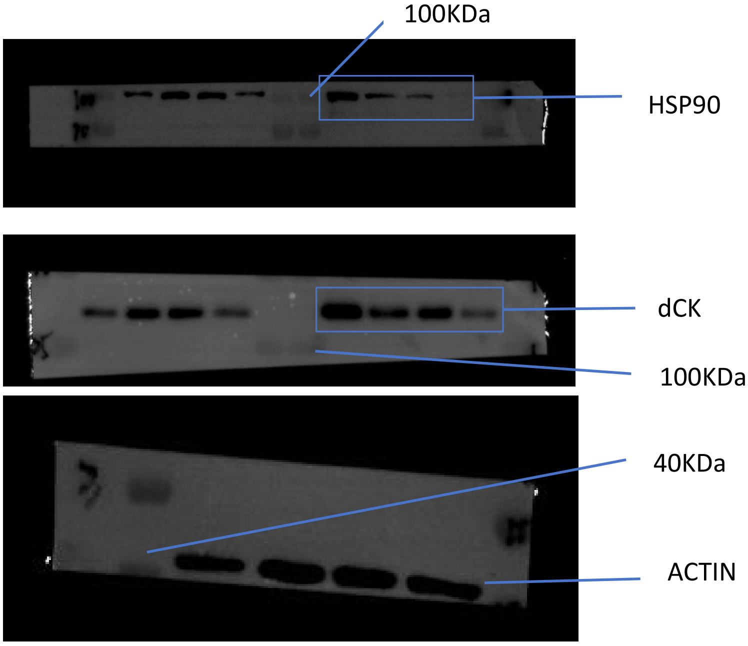

Figure 4E

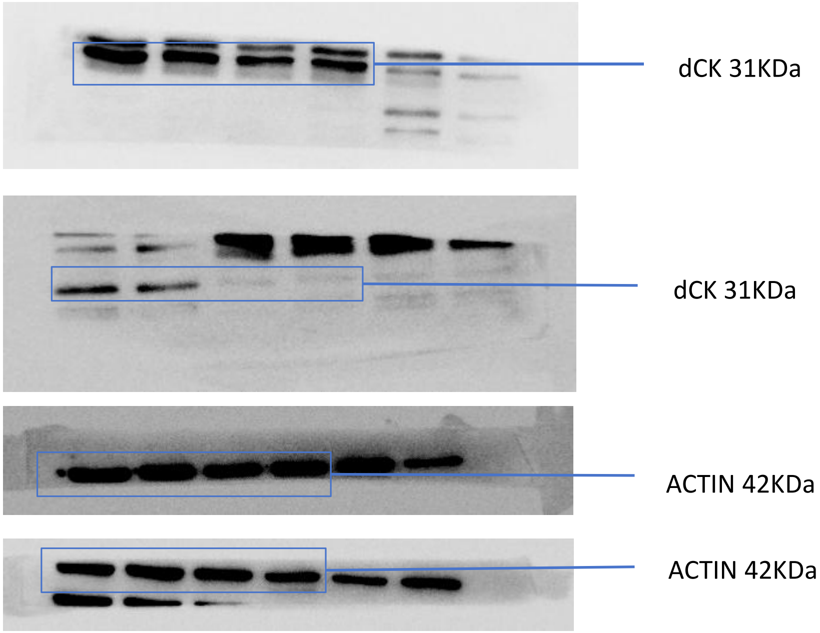

Figure 4G

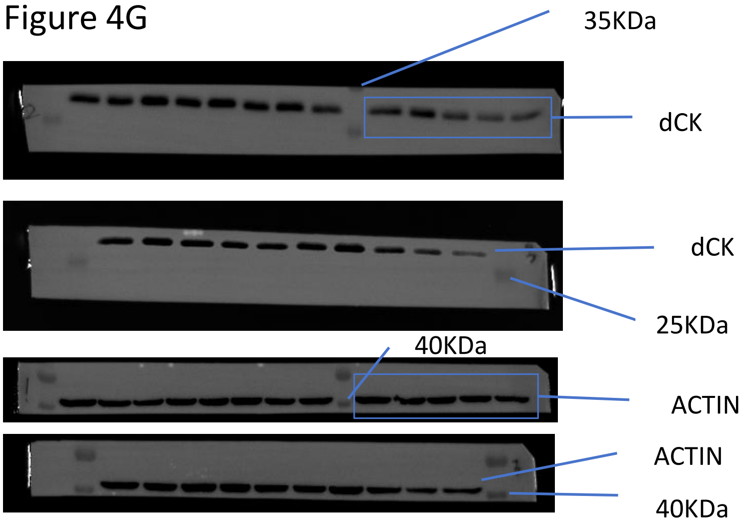

Figure 4I

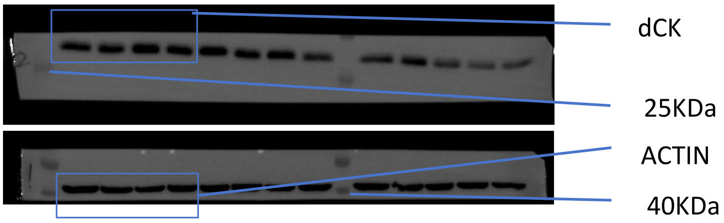

Figure 4J

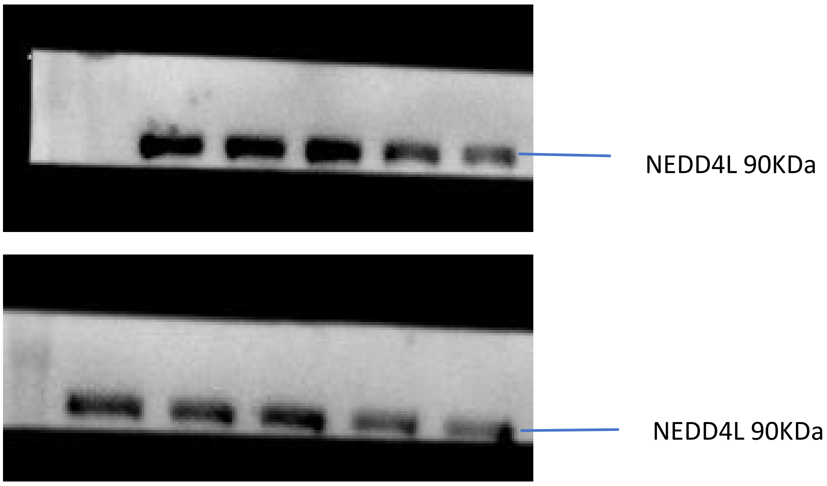

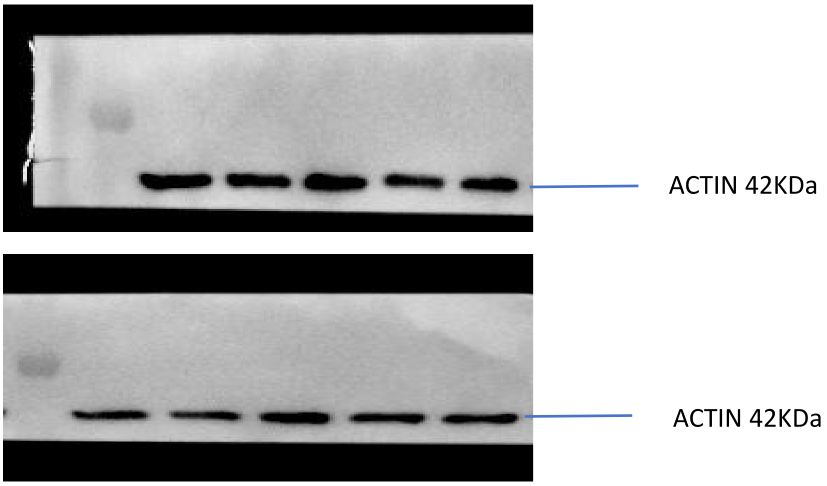

Figure 4L

IP：Flag

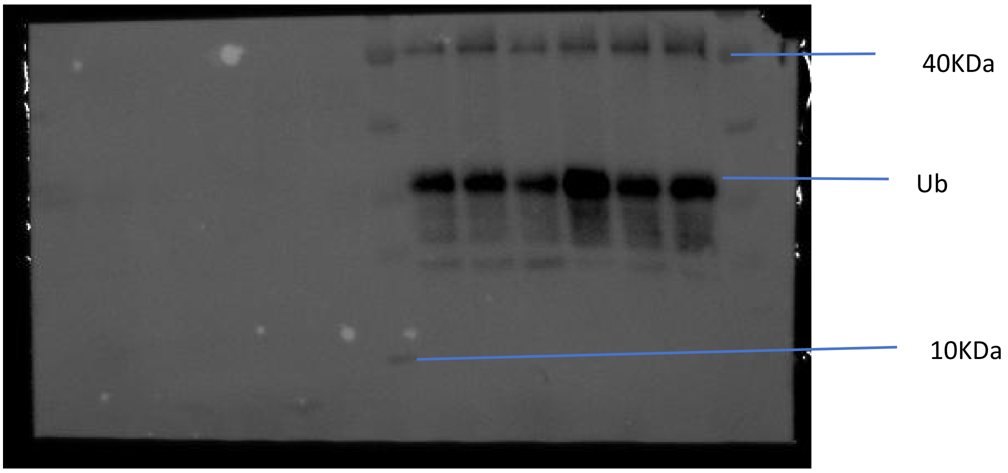

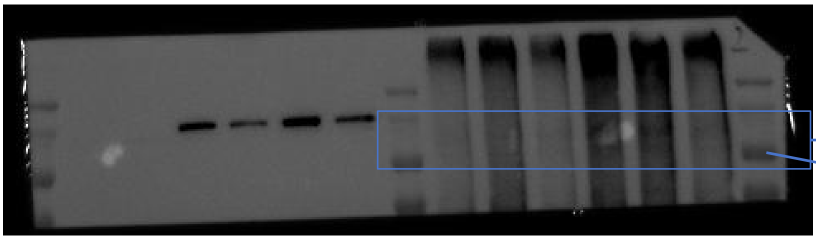

HA

100KDa

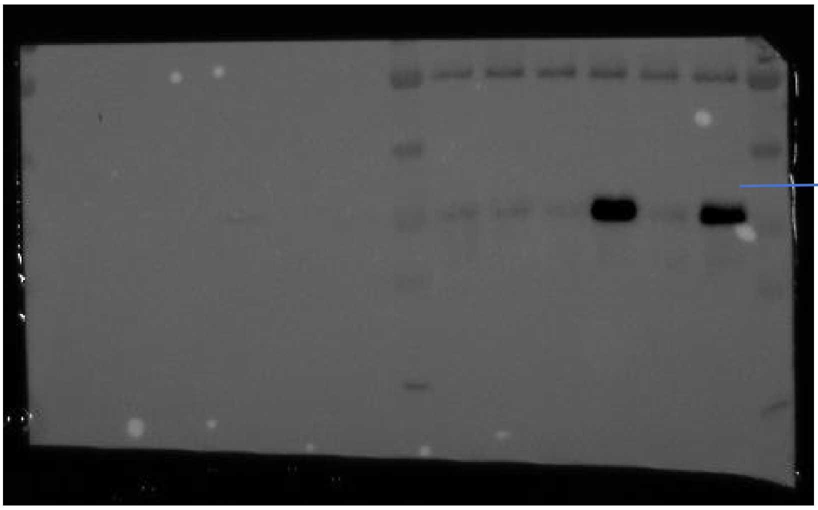

Flag

Input

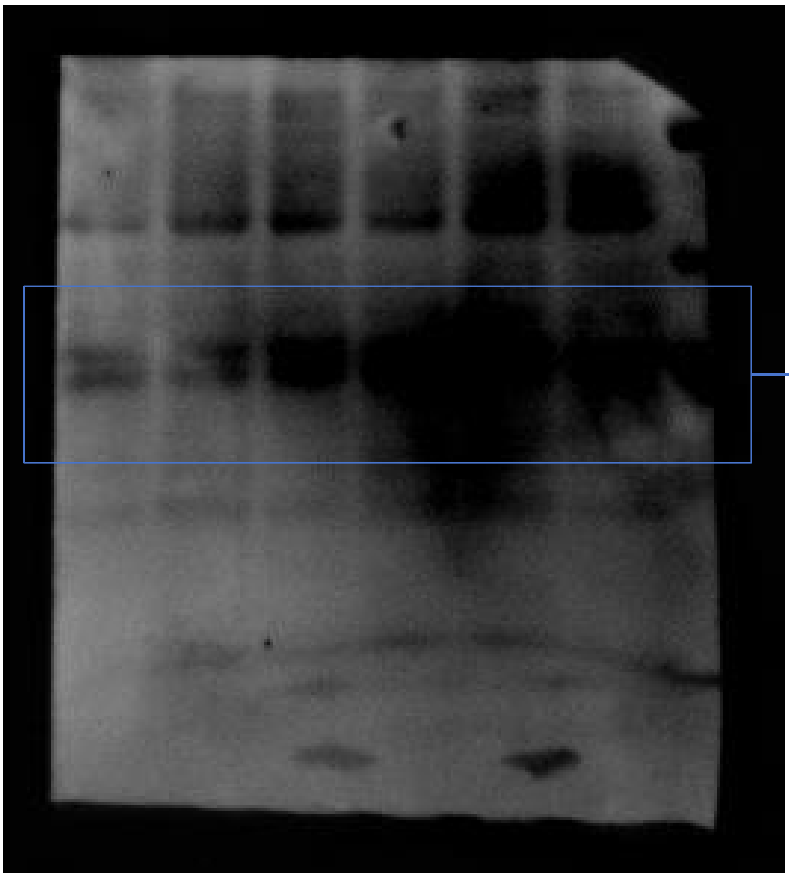

Ub

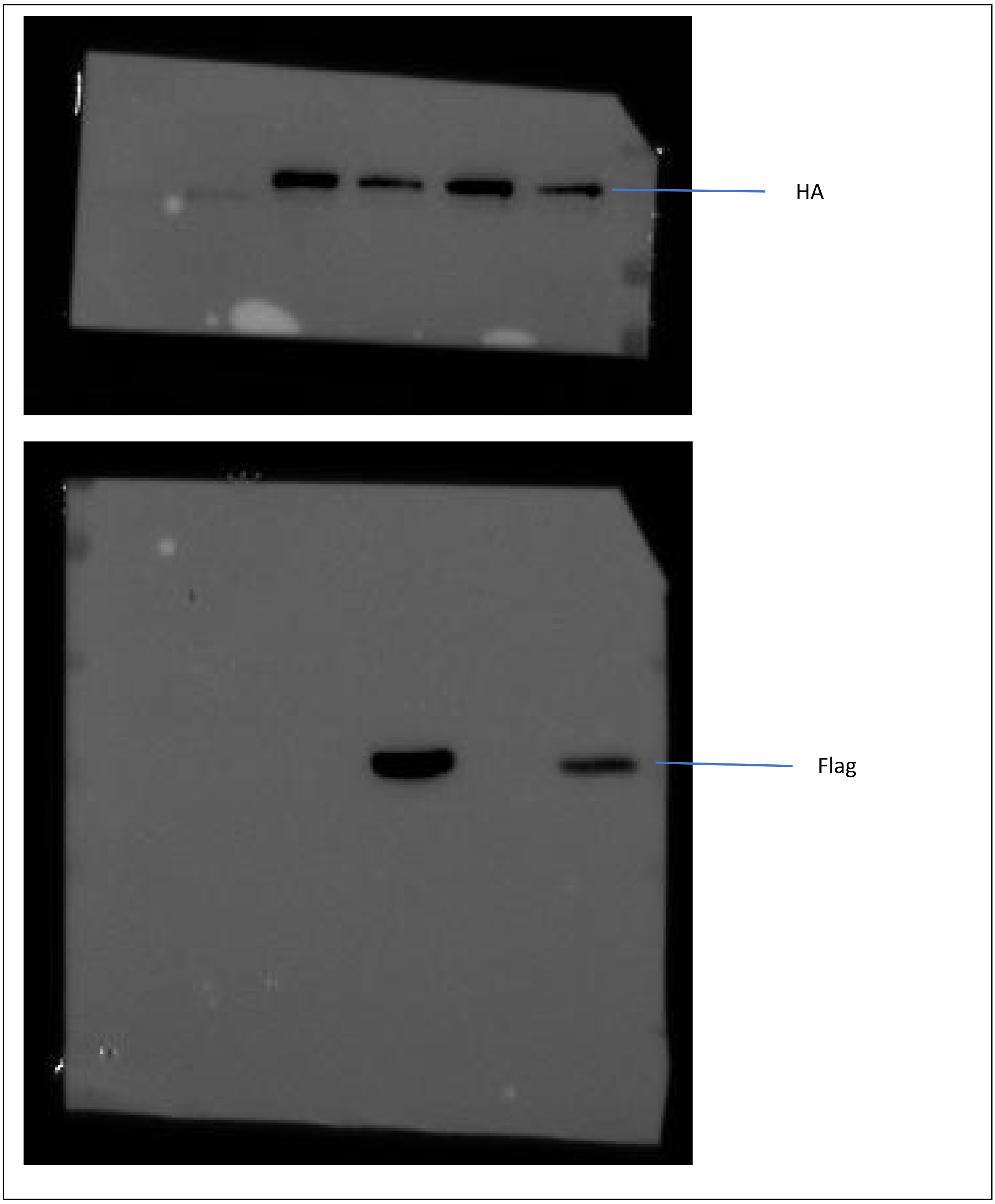
HA
Flag

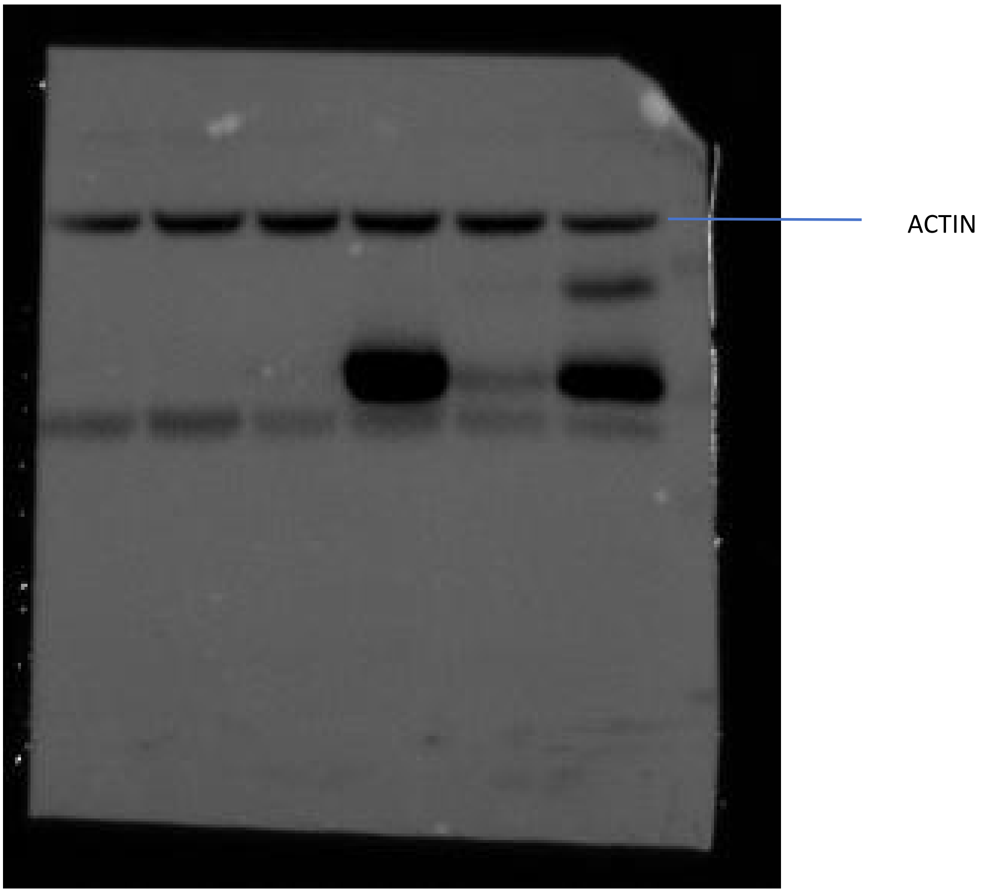

Figure 4M

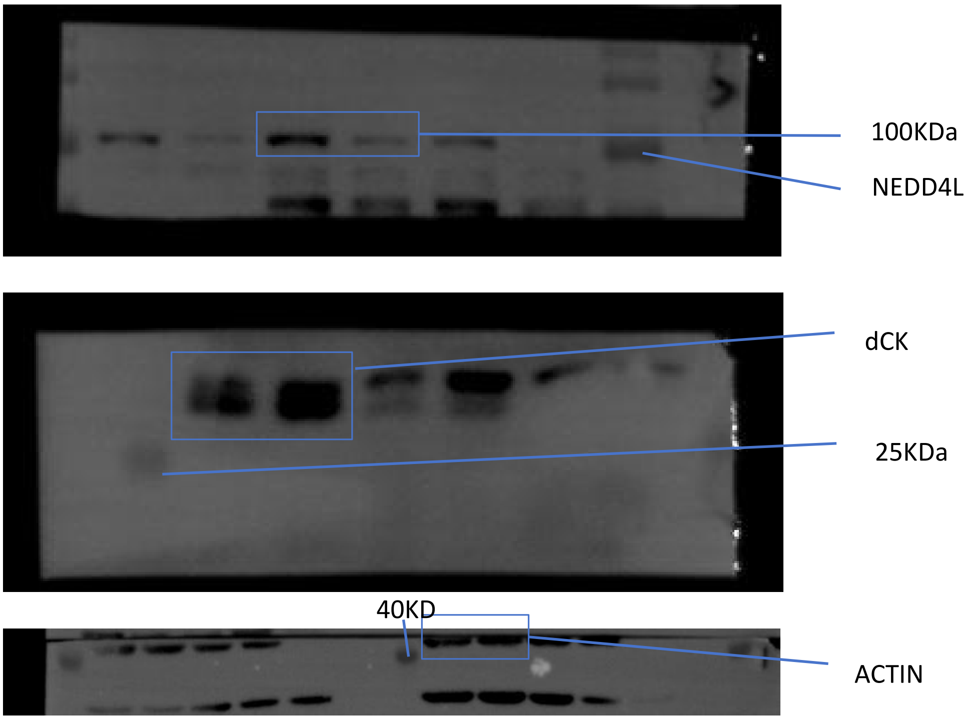

Figure 4N

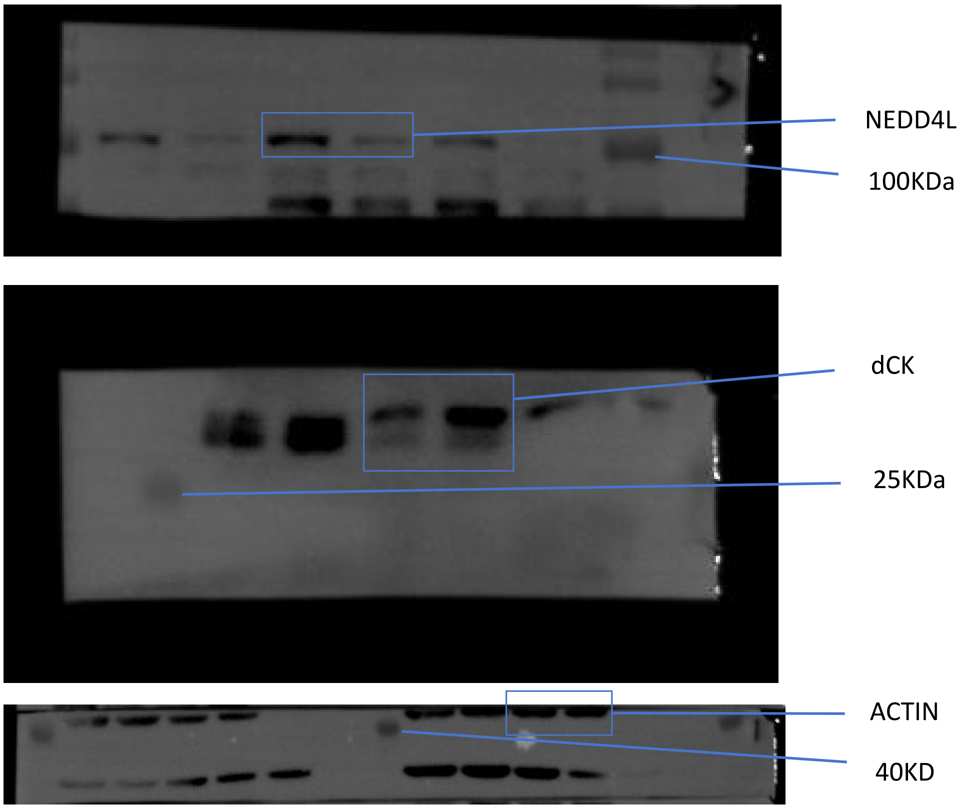

Figure 4O

IP:Myc

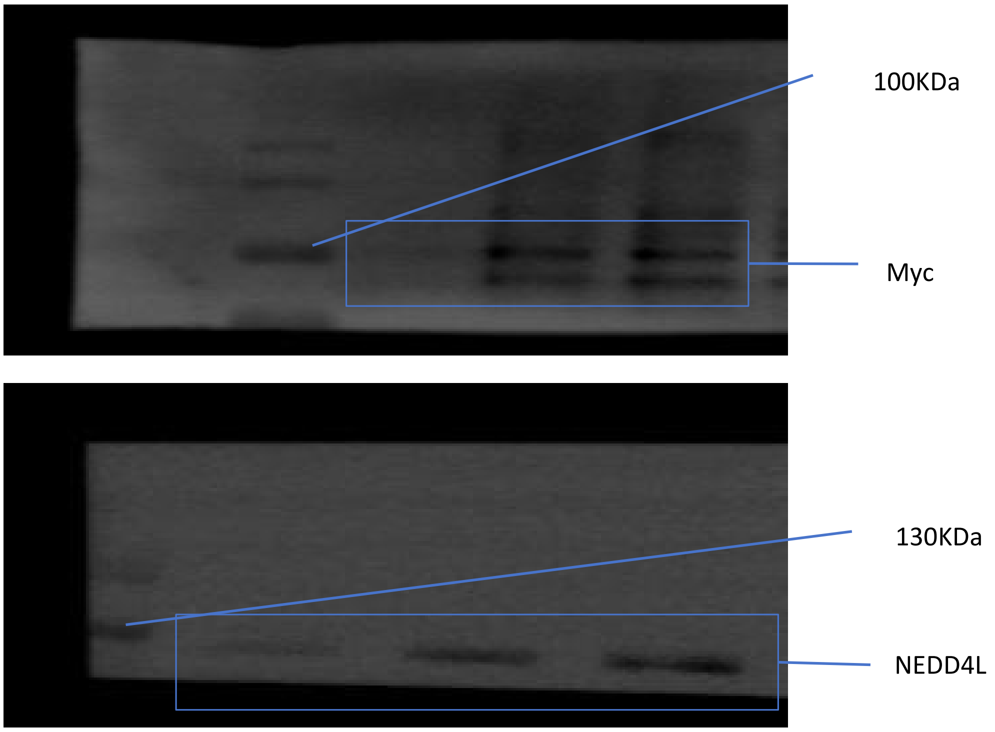

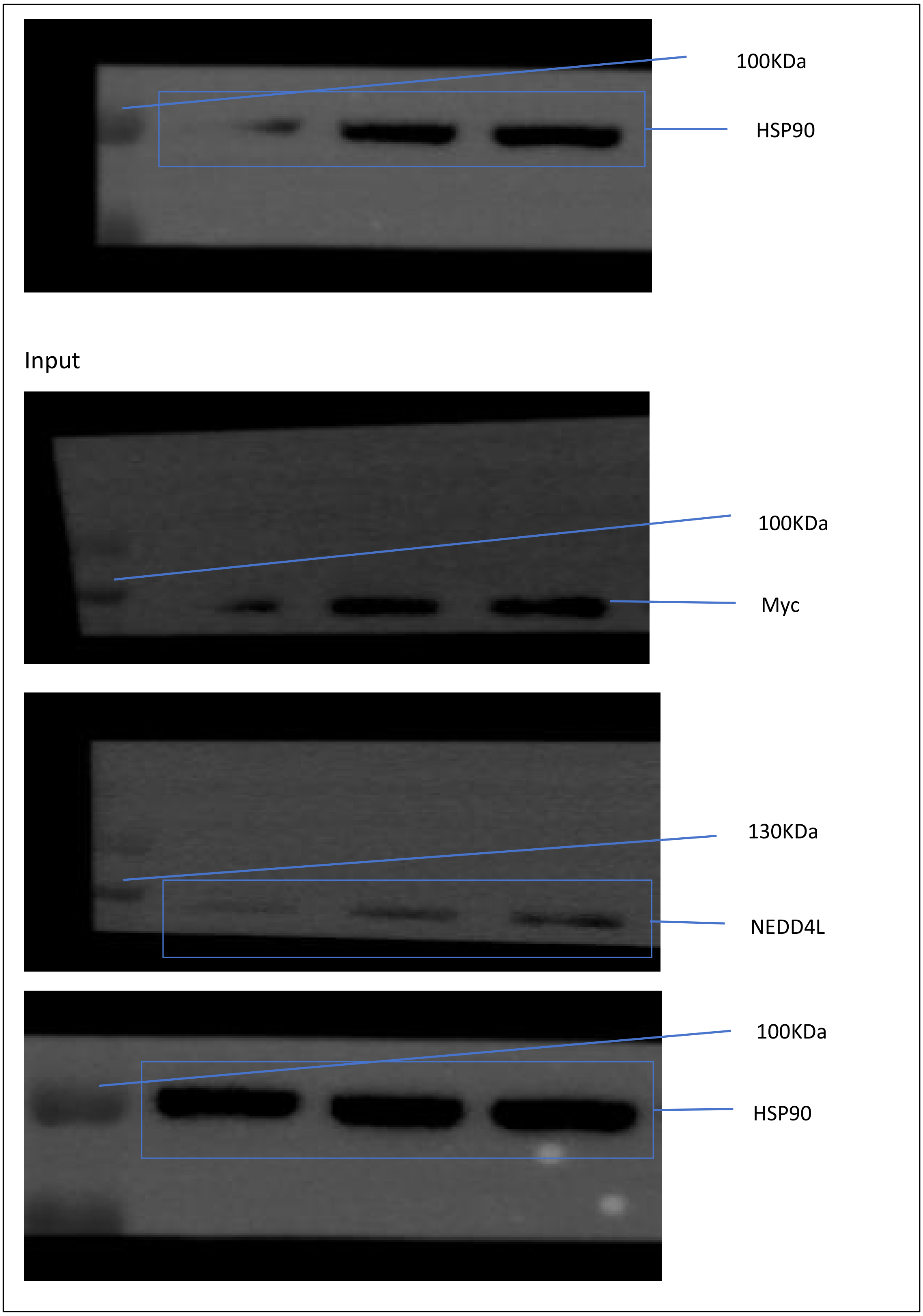
100KDa
HSP90
Input
100KDa
Myc
130KDa
NEDD4L
100KDa
HSP90

Figure 6A

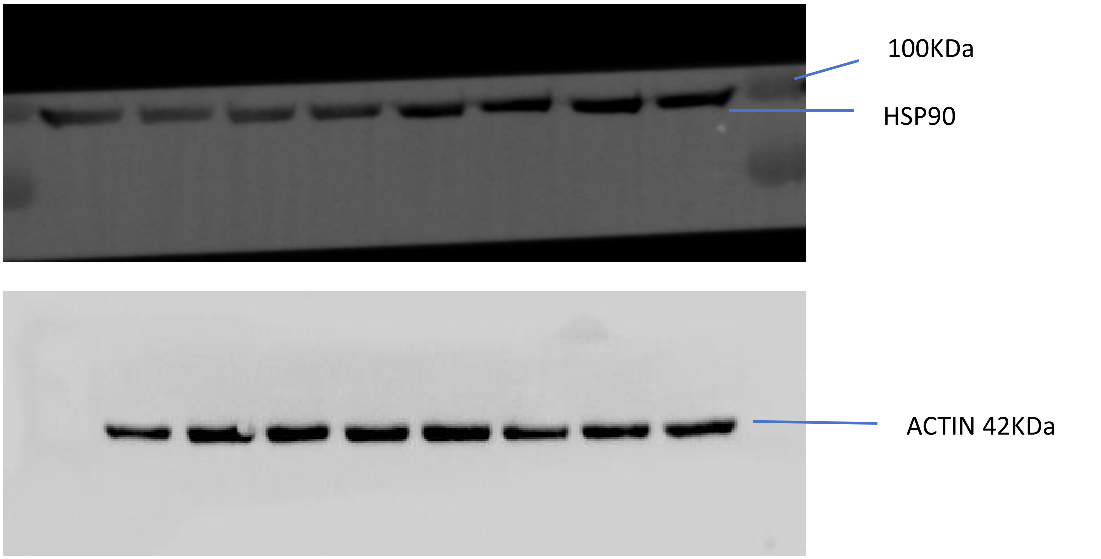

Supplement: Supplementary file 2 — Original Data [file 41420_2025_2388_MOESM2_ESM.pdf]
